# Diesel exhaust particles distort lung epithelial progenitors and their fibroblast niche☆

**DOI:** 10.1016/j.envpol.2022.119292

**Published:** 2022-04-18

**Authors:** Xinhui Wu, Chiara Ciminieri, I. Sophie T. Bos, Manon E. Woest, Angela D’Ambrosi, René Wardenaar, Diana C.J. Spierings, Melanie Königshoff, Martina Schmidt, Loes E.M. Kistemaker, Reinoud Gosens

**Affiliations:** a Department of Molecular Pharmacology, Faculty of Science and Engineering, University of Groningen, Antonius Deusinglaan 1, 9713AV, Groningen, the Netherlands; b Groningen Research Institute for Asthma and COPD, University Medical Center Groningen, University of Groningen, Groningen, the Netherlands; c Aquilo BV, Antonius Deusinglaan 1, 9713AV, Groningen, the Netherlands; d European Research Institute for the Biology of Ageing (ERIBA), University of Groningen, University Medical Center Groningen, 9713AV, Groningen, the Netherlands; e Division of Pulmonary, Allergy and Critical Care Medicine, Department of Medicine, University of Pittsburgh, Pittsburgh, USA

**Keywords:** Air pollution, Lung repair, Organoids, Oxidative stress, WNT signaling

## Abstract

Chronic obstructive pulmonary disease (COPD) is a progressive lung disease characterized by inflammation and impaired tissue regeneration, and is reported as the fourth leading cause of death worldwide by the Centers for Disease Control and Prevention (CDC). Environmental pollution and specifically motor vehicle emissions are known to play a role in the pathogenesis of COPD, but little is still known about the molecular mechanisms that are altered following diesel exhaust particles (DEP) exposure. Here we used lung organoids derived from co-culture of alveolar epithelial progenitors and fibroblasts to investigate the effect of DEP on the epithelial-mesenchymal signaling niche in the distal lung, which is essential for tissue repair. We found that DEP treatment impaired the number as well as the average diameter of both airway and alveolar type of lung organoids. Bulk RNA-sequencing of re-sorted epithelial cells and fibroblasts following organoid co-culture shows that the Nrf2 pathway, which regulates antioxidants’ activity, was upregulated in both cell populations in response to DEP; and WNT/β-catenin signaling, which is essential to promote epithelial repair, was downregulated in DEP-exposed epithelial cells. We show that pharmacological treatment with anti-oxidant agents such as N-acetyl cysteine (NAC) or Mitoquinone mesylate (MitoQ) reversed the effect of DEP on organoids growth. Additionally, a WNT/β-catenin activator (CHIR99021) successfully restored WNT signaling and promoted organoid growth upon DEP exposure. We propose that targeting oxidative stress and specific signaling pathways affected by DEP in the distal lung may represent a strategy to restore tissue repair in COPD.

## 1. Introduction

Air pollution is defined as “contamination of the indoor or outdoor environment by any chemical, physical or biological agent that modifies the natural characteristics of the atmosphere” by the World Health Organization (WHO). Both the ambient (outdoor) and indoor pollution are responsible for the death of an estimated seven million people worldwide every year, as a result of increased mortality from several cardiovascular and pulmonary diseases (World Health Organization, 2021), and therefore air pollution represents a great burden in the contexts of public and occupational health. Sources of ambient pollution include, among others, motor vehicle emissions which produce gaseous air pollutants and particulate matter (PM); the largest source of airborne PM from motor vehicles is derived from diesel exhaust, producing fine (2.5–0.1 μm) or ultrafine (<0.1 μm) particles. The epithelium that lines the respiratory tract represents the first and major target for diesel exhaust particles (DEP); the small size and large surface area of these particles allow them to penetrate and deposit deep into the small airways and alveoli, where they can accumulate and trigger inflammation and oxidative stress resulting in acute or chronic lung injuries (Aghapour et al., 2022; Nemmar et al., 2012; Riedl and Diaz-Sanchez, 2005; Risom et al., 2005; Wichmann, 2007). Normally, the cells are able to counteract DEP-derived inflammation and excessive reactive oxygen species (ROS) production through activation of the Nrf2-antioxidant response element signaling pathway, which promotes the activity of phase II drug metabolizing and antioxidant enzymes, such as NAD(P)H: quinone oxidoreductase (NQO1) and several glutathione-S-transferases (GSTs). However, daily exposure to air pollution may cause a continuous insult that reduces the efficacy of the anti-oxidant defenses, bringing an imbalance which results in cell apoptosis, necrosis, and DNA damage (Cosselman et al., 2020; Long and Carlsten, 2022; Riedl and Diaz-Sanchez, 2005; Steiner et al., 2016).

Environmental and occupational pollution, together with cigarette smoke and/or genetic predisposition are the main causes of chronic obstructive pulmonary disease (COPD), a progressive lung disease characterized by chronic bronchitis, excessive mucus production, small airways remodeling and emphysema. COPD patients are also more susceptible to other insults, such as viral and bacterial infections that cause exacerbations of the disease, leading to decline in lung function and progressively worsening the disease. Several studies found that air pollution exposure was correlated with the incidence of new cases of COPD (Shin et al., 2021) and with decreased lung function and higher COPD prevalence (Doiron et al., 2019; Elbarbary et al., 2020). Exposure to air pollution is also correlated with increased mortality of COPD patients (Hart et al., 2009) and increased incidence rate of severe acute exacerbations of COPD (Choi et al., 2018; Sint et al., 2008).

Emphysema, which is the destruction of alveolar airspaces in the most distal part of the lung where gas exchange occurs, is caused, among other factors, by inflammation and oxidative stress (Agustí and Hogg, 2019). Several studies showed that chronic exposure to DEP can cause airspace enlargement in the alveoli of healthy mice (Yoshizaki et al., 2015), worsen protease-induced emphysema and increase pulmonary remodeling in mice (Lopes et al., 2009; Moreira et al., 2020). The alveoli are comprised of alveolar stem cell niches, where mesenchymal fibroblast cells support neighboring epithelial progenitor cells in homeostasis and injury, controlling their fate through several signaling factors, including growth factors (EGF, FGF) and WNT signaling (Hogan, 2020). In COPD, when emphysema and alveolar tissue destruction occurs, this supporting mesenchymal-epithelial signaling is altered. This causes reduced activation, proliferation and/or differentiation of the epithelial progenitor cells and therefore failed tissue repair.

While there are drugs and standards of care to ease the symptoms of COPD, there are currently no therapies available to slow or halt the progression of the disease, and patients in the late stage have the only option of undergoing transplantation. Understanding the molecular mechanisms involved in impaired lung tissue regeneration could lead to identification of new druggable targets to restore a pro-repair signaling in the lung progenitors’ niche and prevent disease worsening. In this study we used organoids, an established 3D *in vitro* co-culture system, to investigate the effect of DEP exposure on the alveolar progenitor niche, and performed a transcriptome analysis to identify the molecular signaling pathways regulated in both fibroblasts and epithelial cells in response to DEP, thereby discovering potential drug targets to restore endogenous lung repair.

## 2. Materials and methods

### 2.1. Animal experiments

C57BL/6J mice (both genders, aged 8–12 weeks) were maintained in 12 hours light/dark cycles and had access to food and water ad libitum at the Central Animal Facility (CDP) of the University Medical Center Groningen (UMCG). All experiments were done in accordance with Directive 2010/63/EU for animal experiments, the national ethical guidelines and upon approval of the experimental procedures the Institutional Animal Care and Use Committee (IACUC) of the University of Groningen under the CCD license (AVD105002015303).

### 2.2. Reagents preparation

A stock solution from the diesel particulate matter (NIST 2975, Sigma-Aldrich Chemie N.V.) in culture medium was prepared according to the manufacturer’s instructions; details can be found in the Extended materials and methods.

N-Acetyl-L-cysteine (NAC, A7250, Sigma-Aldrich), Mitoquinone mesylate (MitoQ, HY-100116A, MedChemExpress) and CHIR99021 (CHIR, 1386, Axon Medchem) were dissolved in ultrapure water and stored as stock solutions at −20 °C. They were further diluted in culture medium prior to treatment and used in the following concentrations: NAC 1 mM, MitoQ 1 μM, CHIR 2 μM. All other reagents used in the study were of analytical grade.

### 2.3. Organoid culture and immunofluorescence staining

**Fig. 1.**
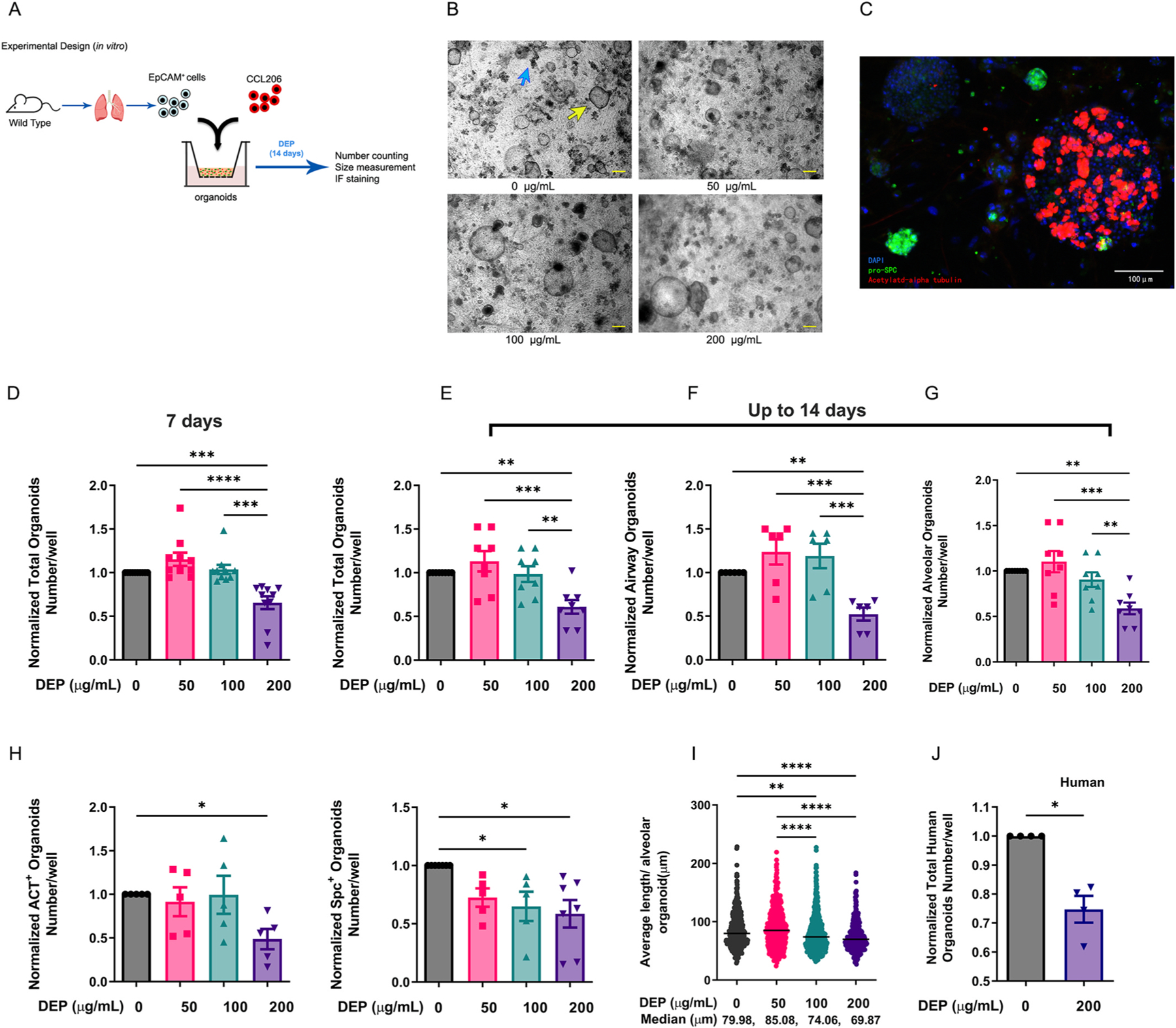
Exposure to diesel exhaust particles (DEP) represses lung organoid formation. **(A)** Schematic of experimental design. **(B)** Representative images of murine lung organoids at day 14 treated with different concentrations of DEP (control, 50, 100, and 200 μg/mL) taken by light microscopy. Blue arrow: alveolar-type organoids; yellow arrow: airway-type organoids. Scale bar = 200 μm. **(C)** Representative image of immunofluorescence (IF) staining of murine lung organoids stained with DAPI (blue), pro-Surfactant C (SPC, green), and acetylated-a tubulin (ACT); scale bar = 100 μm. **(D)** Quantification of normalized total organoids treated with 0 (control), 50, 100, or 200 μg/mL DEP for 7 days. N = 10 **(E**–**G)** Quantification of normalized murine total (**E**, N = 8), airway (**F**, N = 6) and alveolar type organoids (**G**, N = 8) on day 14 after continuous treatment with different concentrations of DEP. **(H)** Quantification of normalized murine ACT+ and SPC + organoids treated with control, 50, 100, or 200 μg/mL DEP. N = 5. **(I)** Comparison of average diameter of murine alveolar type organoids (median value) treated with 0, 50, 100, or 200 μg/mL DEP measured on day 14 (n > 314 organoids/group obtained from N = 5 mice). **(J)** Quantification of normalized total human organoids (N = 4) treated with 0 (control) or 200 μg/mL DEP for 14 days. Data are presented as mean ± SEM. *p < 0.05, **p < 0.01, ***p < 0.001, ****p < 0.0001. (For interpretation of the references to colour in this figure legend, the reader is referred to the Web version of this article.)

The organoid culture system is based on protocols previously published by our group (Ng-Blichfeldt et al., 2019; Wu et al., 2019). For murine organoids, mouse pulmonary Epcam^+^ cells (CD31^-^/CD45^-^/CD326^+^) were freshly isolated from murine lung tissue without the trachea using the QuadroMACS^™^ Separator (Miltenyi Biotec). 20,000 Epcam^+^ cells and 20,000 mouse fibroblasts (CCL-206, ATCC) were mixed and suspended in 100 μL of Matrigel^®^ (354230, Corning) prediluted 1:1 (v/v) with DMEM (Gibco) supplemented with 10% FBS. Next, the cell mixtures were added to 24-well Falcon^®^ cell culture inserts (Corning) in a 24-well plate containing 400 μL of organoid media (DMEM/F-12 with 5% FBS, 1% penicillin/streptomycin, 1% glutamine, 1% amphotericin B, 0.025% EGF, 1% insulin transferrin-selenium, and 1.75% bovine pituitary extract) underneath. Fresh medium and treatments were replaced every 2–3 days. The number of organoids was counted by eye using light microscopy with 20x magnification, and the diameter of the individual organoids (organoid size) was measured using NIS-Elements software (Nikon). Under light microscopy, alveolar and airway-type organoids were recognized by their morphology (Fig. 1B). The detailed protocols for human organoids, immunofluorescence staining and organoids resorting are available in Extended materials and methods.

### 2.4. RNA extraction, RNA sequencing (RNA seq) analysis and quantitative real-time PCR (qRT-PCR)

Total RNA was extracted from A549 cells using TRIzol^™^ (Life Technologies) for qRT-PCR analysis. Total RNA was extracted from cells re-sorted from organoids using NucleoSpin^®^ RNA kit (740955, MACHEREY-NAGEL) according to the manufacturer’s instructions. RNA concentrations and quality were assessed by Nanodrop spectrophotometer (Thermo Fisher Scientific) and Bioanalyzer system (Agilent). Transcriptome analysis was performed in collaboration with the European Research Institute for the Biology of Ageing (ERIBA, Groningen, The Netherlands). The complete protocols for RNAseq and qRT-PCR are described in Extended materials and methods.

### 2.5. DCFDA assay

The DCFDA assay (2′,7’ –dichlorofluorescin diacetate, ab113851, Abcam) was performed to detect reactive oxygen species (ROS) in the cells according to manufacturer’s instructions. Briefly, A549 cells were seeded in a black 96-well plate with clear bottom and then put in starvation medium (0.5% FBS) for 24 hours. Cells were then treated for 24 hours with 200 μg/mL DEP ± NAC or MitoQ in the previously described concentrations. After treatment, cells were washed with PBS and incubated with the DCFDA dye for 45 minutes at 37 °C in the dark. Fluorescence was measured at Ex 485 nm/Em 535 nm with the Synergy HTX Multi-Mode Microplate Reader (BioTek). Data were collected with Gen5 software (BioTek).

### 2.6. TOP/FOP flash assay

The TOP/FOP flash assay was performed according to the protocol previously described by our group (Wu et al., 2019). Briefly, A549 cells were seeded in 96-well plate and transfected with 100 ng/well of either a luciferase reporter plasmid (TOP) or the negative control plasmid (FOP), using Lipofectamine^™^ LTX Reagent with PLUS^™^ Reagent (Invitrogen) in serum-free OptiMEM^®^ medium (Life Technologies). After 5 hours, cells were treated for 24 hours with 200 μg/mL DEP and/or CHIR (2 μM) in Opti-MEM^®^ medium supplemented with 0.1% FBS. Cells were then washed with PBS, lysed using Glo Lysis Buffer (E2661, Promega) and incubated with Bright-Glo^™^ Luciferase Assay System (E2610, Promega). WNT activation was assessed by measuring luminescence using the Synergy HTX Multi-Mode Microplate Reader (BioTek). Data were collected with Gen5 software (BioTek).

### 2.7. Data analysis and statistics

Functional data are presented as mean ± SEM, unless stated otherwise. Statistical significance of differences was evaluated by Student’s T-test or one-way ANOVA, where appropriate. The p-value indicating statistically significant differences between the mean values are defined as follows: *p < 0.05, **p < 0.01, ***p < 0.001, ****p < 0.0001. Statistical analyses were performed with GraphPad Prism 9 software.

## 3. Results

### 3.1. Diesel exhaust particles (DEP) affect lung organoid growth

To investigate the effect of DEP on lung epithelial progenitors, we used an established model of lung organoids derived from the co-culture of primary mouse epithelial (Epcam^+^) cells, which comprise the alveolar progenitor cells, and murine fibroblasts (CCL-206), as depicted in Fig. 1A. We used different concentrations of DEP (50, 100, 200 μg/mL), chosen in reference to previous *in vitro* studies (Durga et al., 2014; Ou et al., 2021) to treat the organoids continuously for up to 14 days to analyze dose-dependent effects. Quantification of the number of organoids at day 7 and 14 (Fig. 1D and E) showed a significant decrease in total number of organoids upon treatment with 200 μg/mL DEP. This decrease was consistent and statistically significant for both airway-type and alveolar-type organoids quantified at day 14 (Fig. 1F and G). Immunofluorescence staining confirmed that the number of acetylated-α tubulin^+^ (ACT^+^, airway) and pro-surfactant protein C^+^ (pro-SPC^+^, alveolar) organoids was significantly decreased by 200 μg/mL DEP (Fig. 1H). The size of alveolar-type organoids at day 14 was also significantly reduced by 100 and 200 μg/mL DEP (Fig. 1I). Comparably, DEP (200 μg/mL) caused a significant decrease in the total number of human lung organoids at day 14 (Fig. 1J). Taken together, these data show that continuous exposure to 200 μg/mL DEP for 7 and 14 days represses murine organoid growth; a similar impairment is also observed in human lung organoids at day 14.

### 3.2. Transcriptomic mechanisms underlying DEP-induced changes in lung organoids

**Fig. 2.**
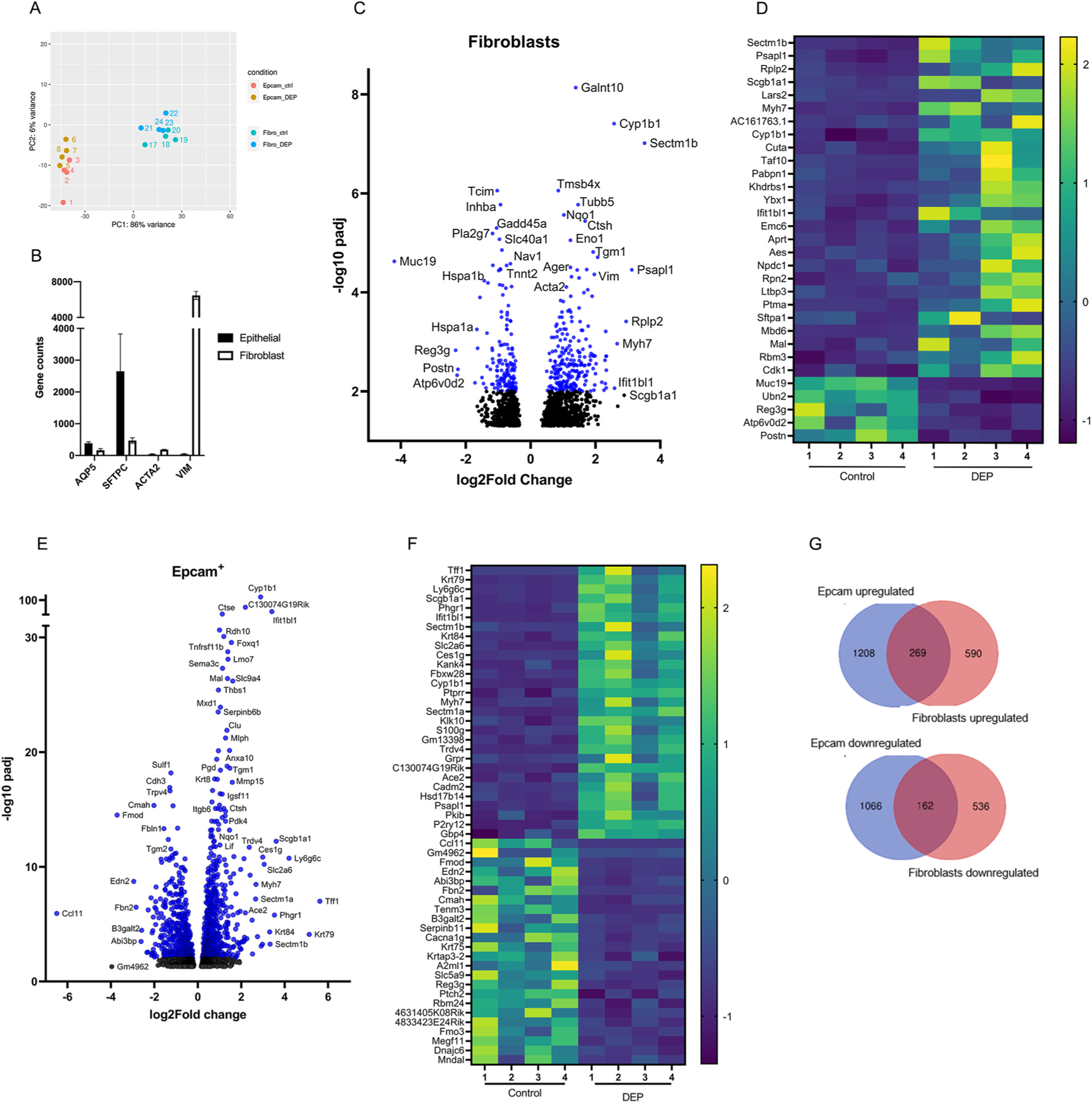
Bulk RNA sequencing of fibroblasts and Epcam + cells resorted from control and DEP-exposed lung organoids shows altered transcriptome in both cell populations. **(A)** Principal component analysis (PCA) demonstrates lung organoid-derived Epcam + cells and fibroblasts cluster separately (N = 4). **(B)** Specific epithelial (Aqp5, Sftpc) and fibroblast (Acta2, Vim) marker gene expression confirms identity and purity of re-sorted Epcam + cells and fibroblasts. **(C)** Volcano plot showing the differentially regulated genes in fibroblasts cells (padj <0.05). Data points shown in blue: padj <0.01; black: 0.05<padj<0.01. **(D)** Heatmap showing the top significant genes which are up- or down-regulated with a log2fold change >2 and < −2 respectively in fibroblasts; data is shown as z-score of the normalized counts for each sample. **(E)** Volcano plot showing the differentially regulated genes in Epcam + cells (padj <0.05). Data points shown in blue: padj <0.01; black: 0.05<padj<0.01. **(F)** Heatmap showing the top significant genes which are up- or down-regulated with a log2fold change >2 and < −2 respectively in Epcam + cells; data is shown as z-score of the normalized counts for each sample. **(G)** Venn diagrams show overlap of up- and down-regulated genes (padj<0.05) between Epcam + cells and fibroblasts re-sorted from DEP-treated organoids (for complete list of genes, see Suppl. Table 1). (For interpretation of the references to colour in this figure legend, the reader is referred to the Web version of this article.)

In order to assess how DEP is affecting the epithelial progenitor niche, after 7 days of 200 μg/mL DEP treatment in the organoids culture, we dissociated the organoids and retrieved the Epcam^+^ cells and CCL-206 fibroblasts to separately subject them to bulk RNA-seq. In Fig. 2A, the principal component analysis (PCA) shows that the two cell population samples were transcriptionally distinct with the primary and secondary components accounting for 86% and 6%, respectively, of the variance across all of the samples. Furthermore, to confirm the cell purity, we compared the normalized gene counts of specific alveolar epithelial and fibroblasts marker genes between these two populations in all untreated samples. As shown in Fig. 2B, *Aqp5* and *Sftpc* were highly expressed in the epithelial population, whereas *Acta2* and *Vim* had a high expression in the fibroblast population. Overall, paired differential expression analysis (DeSeq2) revealed that DEP treatment induced significant upregulation of 859 genes and downregulation of 700 genes in the fibroblasts, and significant upregulation of 1477 genes and downregulation of 1229 genes in the Epcam^+^ population. The differentially expressed genes are represented in function of their significance (padj) and gene expression change (log2fold change) in the volcano plots in Fig. 2C (fibroblasts) and 2E (epithelial cells). Additionally, the z-scores for the top differentially expressed genes are represented in the heatmaps in Fig. 2D and F to show a consistent difference in all our samples between DEP treatment and non-treated control.

In fibroblasts, *Sectm1b* (log2fold change 3.51), *Psapl1* (3.12) and *Rplp2* (2.94) are examples of genes with highest upregulation in DEP compared to non-treated samples, whereas *Muc 19* (log2fold change −4.2), *Reg3g* (−2.3) and *Atp6v0d2* (−2.26) were the most highly downregulated genes (Fig. 2C and D). In Epcam^+^ cells, *Tff1* (log2fold change 5.62), *Krt79* (5.13) and *Ly6g6c* (4.20) had the highest upregulation, while *Ccl11* (log2fold change −6.49), *Gm4962* (−3.96) and *Fmod* (−3.71) had the highest downregulation (Fig. 2E and F). 269 genes were found to be upregulated in both cell types and also 162 genes downregulated (Fig. 2G; Suppl. Table 1); some of these genes were differentially expressed with high statistical significance (padj <0.001) in both cell types. Examples include *Cyp1b1, Nqo1, Aldh3a1* among the upregulated genes*; and Hspa1a* and *Hspa1b* among the downregulated ones, which both code for proteins of the heat shock protein 70 family (Hsp 70). This family of proteins are molecular chaperones involved in protein folding, and they are usually upregulated to protect the cells from stress by correcting protein damage caused by ROS (Gualtieri et al., 2008). Overall, these data show that DEP causes a consistent perturbation in the transcriptome of the cells after 7 days in culture.

**Fig. 3.**
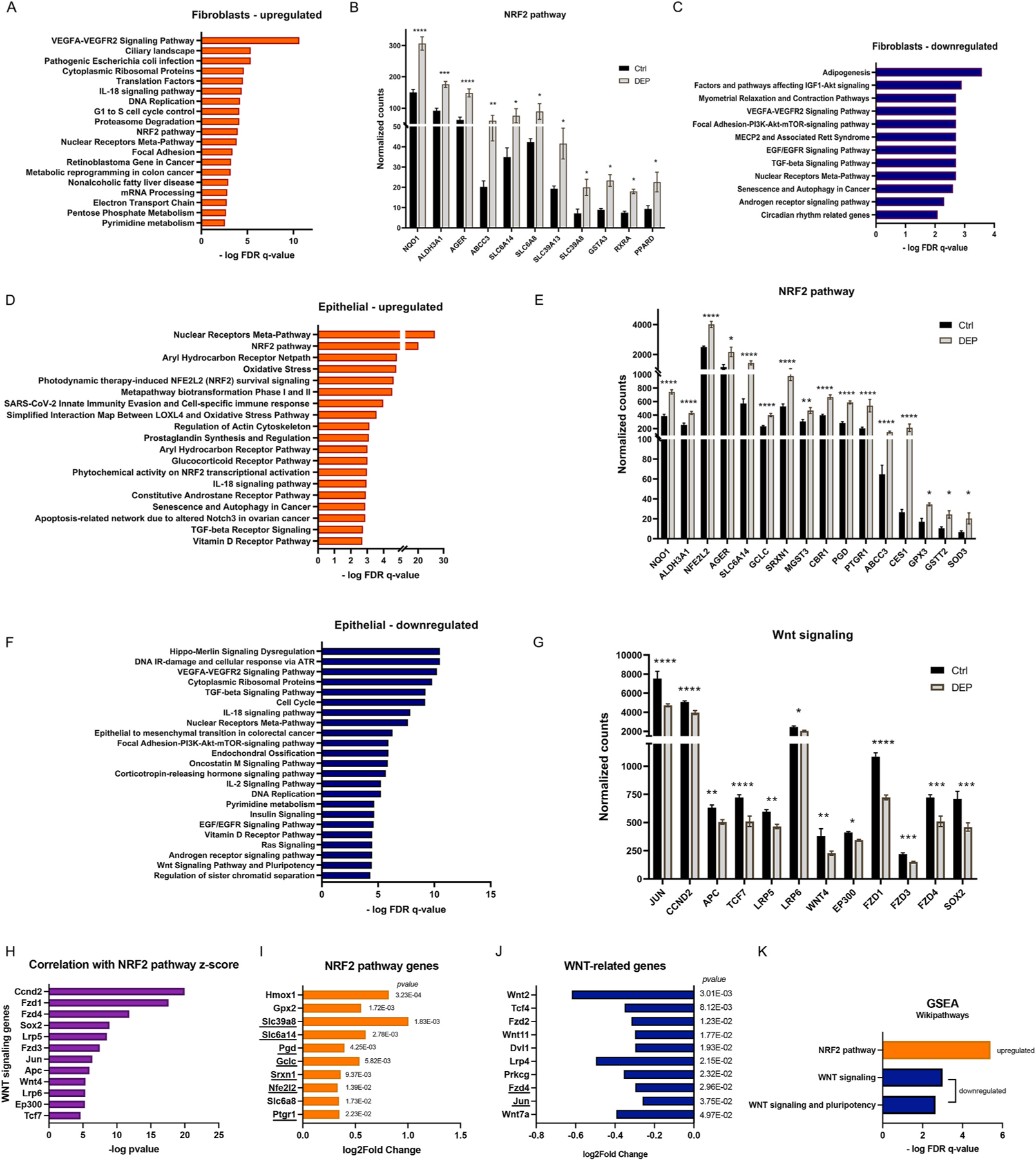
Gene set enrichment analysis (GSEA) identified different molecular signaling pathways in fibroblasts and Epcam + cells derived from organoids exposed to DEP. GSEA (based on WikiPathways gene sets, statistical significance as –logFDR q-value) performed on the top 500 differentially expressed genes (sorted on padj value) in fibroblasts or Epcam^+^ cells re-sorted from organoid treated with DEP. **(A)** Top upregulated pathways in fibroblasts. **(B)** Normalized counts of genes involved in the NRF2 pathway which are upregulated and enriched in fibroblasts. **(C)** Top downregulated pathways in fibroblasts. **(D)** Top upregulated pathways in epithelial (Epcam^+^) cells. **(E)** Normalized counts of genes involved in the NRF2 pathway upregulated in epithelial cells. **(F)** Top downregulated pathways in epithelial cells. **(G)** Normalized counts of WNT-related genes which are downregulated in epithelial cells (Wnt Signaling Pathway and Pluripotency). All pathways represented in the graphs have -logFDR q-value >2. For complete lists of genes, see Suppl. Tables 2-5. **(H)** Correlation of *NRF2 pathway* z-score with genes from the *WNT Signaling Pathway and Pluripotency* in Epcam^+^ cells, statistical significance represented as -log pvalue. **(I–K)** Analysis of an *in vivo* experiment of mice treated with DEP (GEO accession number: GSE22357), N = 3 mice; upregulated genes related to NRF2 pathway **(I),** downregulated genes related to WNT signaling **(J)**, and GSEA showing enrichment of these pathways in the DEP-treated mice samples **(K)**. Underlined genes are commonly regulated in our RNAseq data.

To gain further knowledge into these transcriptomic changes, we performed gene set enrichment analysis (GSEA); using the WikiPathways reference database we identified the molecular pathways over-represented within DEP-modulated genes that are differentially expressed in either epithelial or fibroblast population (top 500 genes with significant up- or down-regulation). In both fibroblast (Fig. 3A) and epithelial (Fig. 3D) populations, DEP treatment caused an enrichment of upregulated genes involved in the *NRF2 pathway*; a representative list of genes involved in this pathway and enriched in either fibroblasts (Fig. 3B) or epithelial cells (Fig. 3E) are shown with their normalized counts in both control and DEP treatment groups. Interestingly, *Nqo1*, *Ager, Aldh3a1, Slc6a14* and *Abcc3* were significantly upregulated in both fibroblasts and epithelial cells treated by DEP. In addition, several signaling pathways were downregulated in both fibroblasts and epithelial cells (Fig. 3C, F), including *TGF-β signaling, Focal adhesion-Akt signaling, VEGFA-VEGFR2 signaling,* and *EGF/EGFR signaling* pathways; all of these pathways share the commonly downregulated genes *Jun, Sos1,* and *Itgb3* (Suppl. Tables 3 and 5). In fibroblasts, we observed that several pathways related to basic functionality and metabolism of the cells, such as cell division (*G1 to S cell cycle control, DNA replication* pathways), *mRNA processing* and *Translation factors* pathways were upregulated (Fig. 3A, Suppl. Table 2). Interestingly, some of these processes (*Cell cycle, DNA replication* pathways) (Fig. 3F) were downregulated in epithelial cells in response to DEP, and several genes, such as the DNA polymerase subunits genes (*Pold1, Pole, Prim 1)* were found downregulated in epithelial cells while upregulated in fibroblasts (Suppl. Tables 2 and 5). In epithelial cells there was a substantial enrichment of genes involved in the metabolization of xenobiotics, as highlighted by the upregulated pathways *Aryl hydrocarbon receptor* and *Metapathway biotransformation phase I and II* (Fig. 3D) which includes several cytochrome P450 enzymes genes (phase I, e.g., *Cyp1b1, Cyp2b6)* and Glutathione S-transferase genes (phase II, e.g. *Gstt2, Gsta3).* Additionally, several genes involved in inflammatory responses, including *Tnf* and *Cxcl6* were also upregulated (Suppl. Table 4). Among the downregulated pathways in epithelial cells (Fig. 3F) we found the *WNT signaling and Pluripotency* pathway; the normalized counts for several representative genes are summarized in Fig. 3G. This includes FZD receptors genes (*Fzd1, Fzd3, Fzd4*), co-receptor genes (*Lrp5, Lrp6),* the histone acetyltransferase gene *Ep300* and the transcription factor *TCF7*. Furthermore, a bioinformatic analysis showed that the WNT signature significantly negatively correlated with the oxidative stress (*NRF2 pathway*) signature in our dataset (pvalue<0.05, Fig. 3H).

To validate our findings with *in vivo* data, we looked for specific gene signatures related to oxidative stress and WNT/β-catenin signaling in a publicly available dataset of microarray data from mice treated with NIST 2975 DEP (GEO accession number: GSE22357) (Stevens et al., 2010). We identified several differentially expressed genes, specifically upregulated genes related to the Nrf2 pathway (Fig. 3I), and downregulated genes related to WNT/β-catenin signaling (Fig. 3J). GSEA confirmed the enrichment of upregulated genes related to the Wiki-Pathways definition *Nrf2 pathway* and enrichment of *Wnt signaling, Wnt signaling and pluripotency* pathways among the downregulated genes (Fig. 3K), showing a correlation of *in vivo* data with our *in vitro* model. We then investigated the effect of DEP in an *ex-vivo* model of murine precision-cut lung slices (PCLS), and confirmed the upregulation of the cytochrome gene *Cyp1b1* and anti-oxidant genes *Nrf2* and *Nqo1* (Supplementary Fig. 1).

Taken together, these data show that treatment of the organoids with DEP affects the fibroblasts and epithelial cells differently in terms of affecting cell homeostasis; however, both cell types exhibit increased oxidative stress. Furthermore, several signaling pathways are downregulated, including pathways involved in tissue homeostasis and regeneration like WNT/β catenin signaling in epithelial cells.

### 3.3. Effects of anti-oxidants NAC, MitoQ and WNT/β-catenin activator CHIR99021

**Fig. 4.**
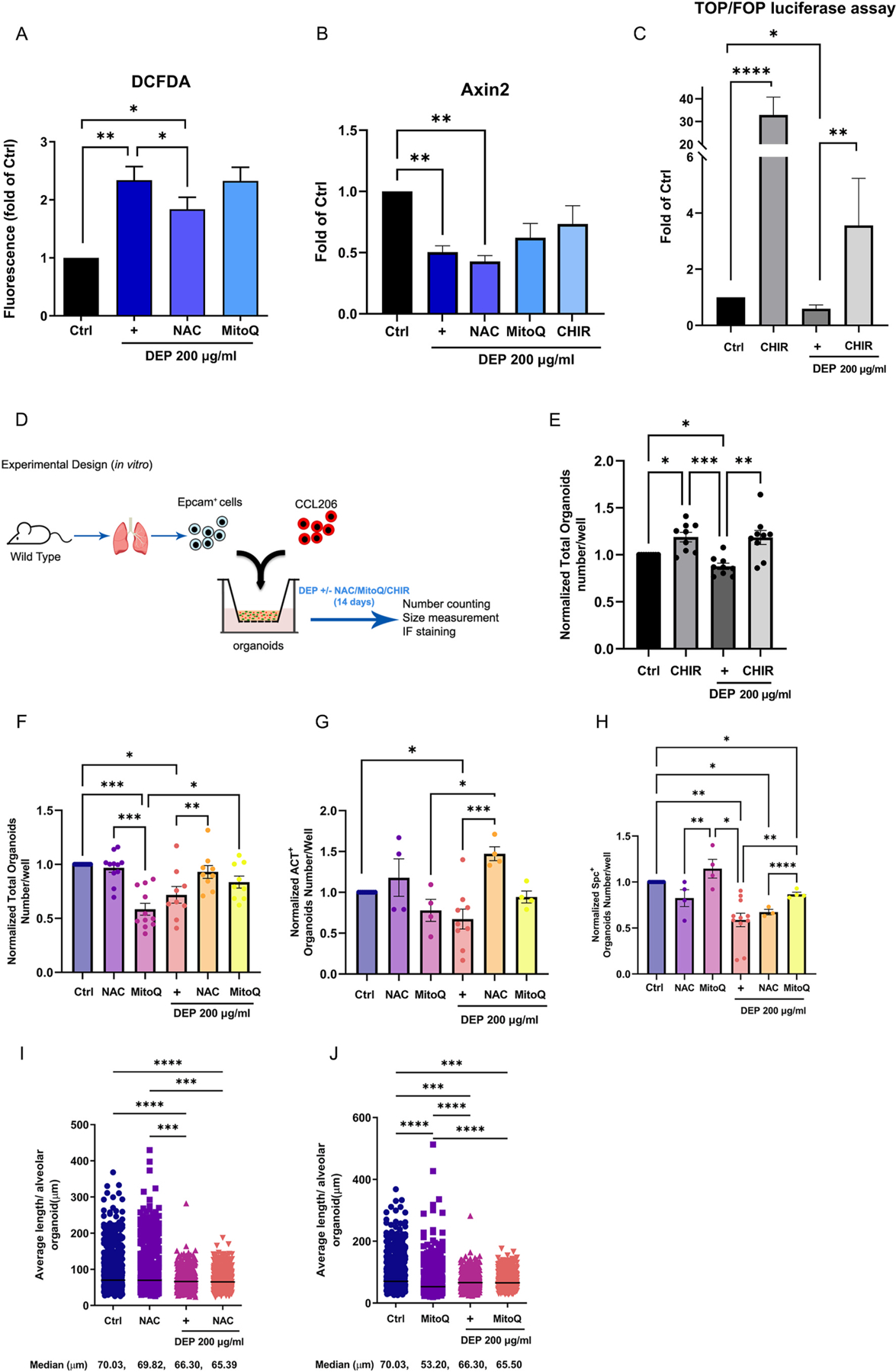
Effects of anti-oxidants (NAC, MitoQ) and WNT signaling activation on the impaired functionality of the epithelial cells and fibroblasts upon DEP exposure. **(A)** DCFDA assay to detect cellular ROS in A549 cells treated with DEP (200 μg/mL) alone or in the presence of NAC (1 mM) or MitoQ (1 μM), N = 6. **(B)** Gene expression of β-catenin target gene Axin2 in A549 cells treated with DEP (200 μg/mL) alone or in the presence of NAC (1 mM), MitoQ (1 μM) or CHIR99021 (CHIR, 2 μM), N = 6. **(C)** TOP/FOPflash assay to determine WNT/β-catenin activation in A549 cells treated with DEP (200 μg/mL) ± CHIR (2 μM), N = 8. **(D)** Schematic of organoids experimental design. **(E)** Quantification of normalized total organoids number after treatment with DEP (200 μg/mL) ± WNT/β-catenin activator CHIR (2 μM). N = 8. **(F)** Quantification at day 14 of normalized total organoids number after treatment with DEP (200 μg/mL), NAC (1 mM), MitoQ (1 μM) and combinations of DEP ± NAC or MitoQ. N = 9–11. **(G**–**H)** Quantification at day 14 of normalized ACT+ (N = 4–9) and SPC+ organoids (N = 3–11) respectively, treated with DEP (200 μg/mL), NAC (1 mM), MitoQ (1 μM) and combinations of DEP ± NAC or MitoQ. **(I)** Quantification at day 14 of average length (diameter) of alveolar type organoids (median value) treated with NAC, DEP (200 μg/mL) or DEP ± NAC; **(J)** or treated with MitoQ, DEP or DEP ± MitoQ (n > 688 organoids/group obtained from N = 9–11 mice). Data are presented as mean ± SEM. *p < 0.05, **p < 0.01, ***p < 0.001, ****p < 0.0001.

Considering the enhanced oxidative stress signature observed in both cell types in response to DEP, we explored the role of anti-oxidants N-acetyl cysteine (NAC) or Mitoquinone (MitoQ) in response to DEP on alveolar epithelial cells; we used A549 (alveolar type II) cells, which have been previously used to study air pollutants and DEP (Ou et al., 2021; Wang et al., 2021). To identify the ROS production, the A549 cells were stained with DCFDA after treatments of DEP (200 μg/mL) ± NAC (1 mM) or MitoQ (1 μM). Compared to control, DEP (200 μg/mL) significantly increased the total cellular ROS level with stronger fluorescence signal (Fig. 4A); this effect was significantly reduced by addition of NAC (1 mM), although the ROS levels were still significantly higher than control. Adding MitoQ did not affect the ROS levels in response to DEP. When looking at gene expression in the treated A549 cells, qRT-PCR (Fig. 4B) showed significant downregulation of β-catenin-target gene *Axin2* in DEP-treated A549 cells (mean 0.504 fold decrease compared to non-treated control), confirming the downregulation of WNT/β-catenin signaling we observed from GSEA analysis of Epcam^+^ cells (Fig. 3F); this effect was unchanged when co-treating with anti-oxidants, and partially rescued by addition of the potent WNT/β-catenin activator CHIR99021 (CHIR) (mean 0.734 fold decrease of control). We further confirmed the effect of DEP on WNT/β-catenin signaling functionally with the TCF/LEF luciferase reporter assay TOP/FOPflash (Fig. 4C). DEP treatment showed significant decrease in WNT activation compared to non-treated control; CHIR alone showed a significant WNT/β-catenin activation as expected (mean 32.95 fold increase of control), and when added to the DEP treatment was able to rescue the WNT/β-catenin activation, although with a fold increase not as high as its effect alone.

Moreover, in the murine lung organoids assay (Fig. 4D and E), CHIR significantly increased the total number of organoids, and this effect was retained even in the presence of DEP, rescuing the impaired organoid formation caused by the diesel exhaust particle treatment. Finally, to explore the effect of anti-oxidants, we treated the murine lung organoids with DEP (200 μg/mL) ± NAC (1 mM) or MitoQ (1 μM) (Fig. 4D, F-J). Fig. 4F shows that at day 14, NAC was able to restore the total number of organoids when added in co-treatment with DEP. MitoQ, on the other hand, impaired organoids formation when added alone, but restored organoids number in co-treatment with DEP. Immunofluorescence staining at day 14 shows significant upregulation of ACT^+^ airway organoids when NAC was added to DEP in treatment compared to DEP alone, while the effect of MitoQ was not significant (Fig. 4G). Regarding SPC^+^ alveolar organoids, MitoQ alone produced a higher ratio of SPC^+^ organoids compared to NAC alone, and MitoQ was able to significantly rescue the impairment caused by DEP (Fig. 4H). Additionally, DEP significantly reduced the size of alveolar organoids, and addition of NAC or MitoQ did not affect this result (Fig. 4I and J). Collectively, these data show that by restoring WNT activation we could revert the impairment on organoids formation caused by diesel exhaust particles. Furthermore, anti-oxidants like NAC and MitoQ can restore the diesel exhaust particles effect on organoids growth, although with different effects on their phenotype.

## 4. Discussion

Most studies on air pollution explore health effects at organ level or systemic level, focusing, for example, on the role of oxidative stress and inflammation on tissue injury. In the present study, we aimed to investigate the effect of diesel exhaust particles specifically on epithelial progenitor cells in the lung, and to identify the molecular mechanisms that are altered in the epithelial progenitor cell niche, thus contributing to failed repair. We used an organoid model derived from lung epithelial progenitors, which are co-cultured with lung fibroblasts in a 3D environment, and their growth was followed over 14 days upon DEP treatment. We observed a dose-dependent effect of DEP on organoid growth, with a significant reduction in total organoid number, size, and specific alveolar (SPC^+^) and airway (ACT^+^) organoid number upon treatment with 200 μg/mL DEP. These results show that accumulation of diesel exhaust particles, even for a relatively short time period (at day 7) can impair the capacity of alveolar epithelial progenitors to form organoids, and the deleterious role of DEP lasts to day 14, resulting in less differentiated types (airway/alveolar) of organoids.

To understand the molecular mechanisms underlying the impaired organoids formation upon DEP treatment, we resorted the epithelial cells and fibroblasts after 7 days in organoid culture and performed sequencing of the full transcriptome. Overall, we were able to identify a substantial number of genes that were significantly down- or upregulated in response to DEP treatment in either cell types, showing that accumulation of diesel particles is able to perturb the homeostasis of lung cells. When we clustered the differentially expressed genes to find their function, we saw that some basic processes of the cell, such as DNA metabolism and cell proliferation were altered, possibly as a result of the increased oxidative stress, and signaling pathways related to epithelial and mesenchymal cells were also affected. Interestingly, the GSEA data shows that while fibroblasts increase the expression of genes related to cell cycle and DNA replication, the opposite happens in epithelial cells, suggesting a different response to the DEP insult.

In both DEP-exposed cell populations, we observed upregulation of the Nrf2 pathway and increased gene expression of *Nqo1* and *Nfe2l2*. This is a physiological response to the DEP-induced oxidative stress, as Nrf2 (*Nfe2l2*) is a transcription factor that controls the expression of genes whose protein products promote detoxification and elimination of reactive oxidants through conjugative reactions, enhancing cellular antioxidant capacity (Nguyen et al., 2009). Activation of Nrf2-mediated anti-oxidant response in the lung upon DEP exposure has been previously described in several studies in mice (Cattani-Cavalieri et al., 2019; Zheng et al., 2019). Additionally, it has been shown that anti-oxidants, such as dimethyl fumarate and emodin, effectively reduce lung injury, inflammation, and oxidative stress induced by chronic DEP exposure in mice (Cattani-Cavalieri et al., 2020; Nemmar et al., 2015). In our studies, we chose two anti-oxidants that are already used as therapeutics, N-acetyl cysteine (NAC) and Mitoquinone (MitoQ), and found they were able to reverse the DEP effect on lung epithelial progenitors. NAC is a potent reducing agent, acting as a precursor of the antioxidant glutathione (GSH) and as a direct ROS scavenger; it is already administered in COPD patients for its known mucolytic, antioxidant and anti-inflammatory properties (Sadowska et al., 2006). MitoQ protects against mitochondrial oxidative damage thanks to its rapid uptake and accumulation in the mitochondria (Smith and Murphy, 2010); it has been proven effective in reducing ROS in models to study lung inflammation (Chen et al., 2019; Wiegman et al., 2015), although its effect on DEP-induced ROS in the lung has not been investigated yet. Therefore, we confirmed production of ROS in a lung epithelial cell line (A549) upon exposure to DEP, as reported by other studies (Patel et al., 2011), and this effect was partially rescued by NAC, but not affected by MitoQ. We then sought to explore the effect of these anti-oxidants on the DEP-treated murine lung organoids. Interestingly, the two drugs had a different effect: NAC in co-treatment with DEP was able to significantly restore the total number of organoids, with a specific effect on airway (ACT^+^) organoids; on the other hand, MitoQ alone seems to have a negative effect on organoids growth, but when added in co-treatment with DEP it showed a significant upregulation of alveolar (SPC^+^) organoids, with no effect on the airway ones. This difference seems to suggest that the harmful effect on alveolar progenitor cells (SPC^+^) may be dependent on mitochondrial ROS, while the airway epithelial cells are more susceptible to cellular ROS; it will be of interest to understand how ROS production regulates different epithelial cell populations. Overall, targeting oxidative stress using antioxidants would be a promising strategy to attenuate lung alveolar epithelial damage induced by DEP; however, the balance with endogenous redox signaling needs to be preserved.

Epithelial cells show enrichment of genes involved in phase I and II of xenobiotics metabolization (Enlo-Scott et al., 2021; Zhang et al., 2006), confirming that this process is active not only in the liver but also in the lung, which is the first site of encounter for inhaled pollutants. *Cyp1b1*, a member of the cytochrome P450 family which is involved in DEP metabolism (Yamazaki et al., 2000), is one of the genes commonly and significantly upregulated in both fibroblasts and epithelial cells. Expression of *Cyp1b1* gene in the lung has been shown before (Kamata et al., 2018; Kim et al., 2004), including upon DEP exposure (Hatanaka et al., 2001). Its expression is dependent on aryl hydrocarbon receptor (AhR) activation (Jacob et al., 2011), and several genes enriched in this pathway were found upregulated in the epithelial cells by GSEA. Among the genes upregulated in both fibroblasts and alveolar epithelial cells in response to DEP in relation to oxidative stress there was *Ager,* which codes for the Receptor for Advanced Glycation End Products (RAGE). RAGE is known to be upregulated in COPD (Wu et al., 2010) and its inhibition enhanced lung repair in various COPD models (Pouwels et al., 2021). Additionally, transgenic mouse models that conditionally over-expressed RAGE in their alveolar epithelium displayed airspace enlargement (Stogsdill et al., 2013) and RAGE activation impaired the alveolar progenitors to form organoids (Pouwels et al., 2021). These results indicate that RAGE might be a potentially interesting target to study in the future for lung repair in response to DEP. Since diesel exhaust particles include many different components, such as a carbon core particle, gaseous components, metals, polycyclic aromatic hydrocarbons (PAHs) etc. (Wichmann, 2007), several studies have tried to understand their contribution to the diesel exhaust toxic effects (for example, Rossner et al., 2016; Wang et al., 2021). We tested the effect of the two PAHs with highest mass fraction in our diesel exhaust particles (1-Nitropyrene, Fluoranthene) and the core carbon particle on murine precision-cut lung slices (PCLS) but did not find any significant effect on some of the genes regulated by DEP in our organoids (data not shown),. A follow-up study, incorporating more of the individual components, alone and in combination will be required to answer in full which of the subcomponents are responsible for which of the functional biological responses.

Emerging evidence has shown that WNT signaling pathway plays an important role in progenitor cells functions, including self-renewal and differentiation during homeostasis and tissue repair (Hu et al., 2021; Raslan and Yoon, 2020). Distinct populations of alveolar progenitor cells responding to WNT signaling upon lung injury and in homeostasis have been described in both mouse (Nabhan et al., 2018) and human (Zacharias et al., 2018) alveoli. In the DEP-exposed epithelial cells we found downregulation of WNT/β-catenin signaling, which may affect the response of the alveolar epithelial progenitor cells upon DEP exposure, contributing to the impaired organoid growth we observed. While we could not confirm these changes at protein level for technical reasons as the organoids provide small amounts of protein for Western blot analyses, we were able to confirm the effect of DEP on WNT/β-catenin signaling functionally with the TCF/LEF luciferase reporter assay TOP/FOPflash. Additionally, we found a similar WNT downregulation, accompanied by an oxidative stress signature, also in microarray data from mice exposed to DEP, showing these alterations are also present *in vivo.* Distortion of WNT signaling in the lung caused by other toxic particles, such as cigarette smoke has been previously described (Heijink et al., 2013; Wang et al., 2011), while in this study we report for the first time an effect on WNT/β-catenin signaling in lung epithelium caused by diesel exhaust particles. When we tried to restore WNT/β-catenin signaling ectopically using CHIR99021 in the organoids assay, we observed a significant increase in organoids number, and this activation was also capable of restoring the impaired organoids formation caused by DEP. This is in line with previous studies from our group, where we showed that WNT/β-catenin signaling is required to initiate organoids formation in distal lung epithelial progenitors from healthy lung (Hu et al., 2020), while imbalanced WNT signaling affects the fibroblasts’ ability to support alveolar progenitor cells (Wu et al., 2019). Ectopic activation of WNT/β-catenin signaling has also been shown to attenuate experimental emphysema (Cui et al., 2018; Kneidinger et al., 2011) and to allow the impaired epithelial progenitors from experimental emphysema lungs to form organoids (Hu et al., 2020); it has therefore been proposed as a potential therapeutic approach in COPD, although WNT/β-catenin signaling can also be involved in other pathologies or tumor progression, and therefore a targeted and/or a temporally restricted activation needs to be considered. Interestingly, Cui et al. observed that ectopic WNT activation does not attenuate experimental emphysema in Nrf2−/− mice suggesting that Nrf2, at least partially, mediates the protective role of WNT activation against the development of emphysema (Cui et al., 2018).

It will be of interest to identify the mechanisms that link DEP exposure to reduced WNT/β-catenin signaling. In the present study, we report the downregulation of the *Fzd4* gene, which codes for a WNT signaling receptor of the Frizzled (FZD) family; FZD_4_ was previously found downregulated in human and experimental mouse COPD tissue, and especially in primary human alveolar type II (ATII) cells from patients with COPD, together with reduced WNT/β-catenin signaling. Inhibition of FZD_4_ receptor also caused reduced lung organoids formation, which is consistent with our observation (Skronska-Wasek et al., 2017). The underlying mechanisms on the dysfunctional alveolar epithelial progenitors in response to DEP may be mediated by WNT/β-catenin/FZD4 signaling. Additionally, multiple studies (reviewed by Schneider et al., 2014) have shown that the aryl hydrocarbon receptor (AhR) pathway, which can be activated by environmental chemicals and is upregulated in our DEP-exposed Epcam^+^ cells, can negatively regulate WNT/β-catenin signaling pathway in several animal and human models and with different mechanisms of action; a similar interference may be hypothesized in our model, and it could be of interest to assess DEP-induced cross-talk of AhR and WNT in the lung. Interference between mitochondrial ROS, Nrf2 and WNT/β-catenin signaling has also been previously reported in COPD (and reviewed by Qu et al., 2019); mechanistically, we illustrate for the first time a ROS/NRF2/WNT signaling pathway as a potential mechanism for lung repair in response to DEP.

## 5. Conclusion

In conclusion, we show that diesel exhaust particles cause impaired regeneration in a lung organoid model; based on our results, we hypothesize that repeated exposure to DEP, as it happens in everyday exposure in a person’s lifetime, and its accumulation may cause a continuous oxidative stress response which could become harmful if not counteracted by anti-oxidant therapeutic agents. Moreover, we report a distortion in epithelial and mesenchymal signaling, and we conclude that, among other factors, reduced WNT signaling may contribute to failed repair, and ectopic WNT activation may represent a feasible strategy to restore a pro-regeneration microenvironment in the distal lung. Further studies in *in vivo* models and in COPD patients will be needed to confirm these hypotheses.

## Supplementary Material

Appendix

Supplement

## Data Availability

All relevant data are included in the manuscript and supporting information and are also available from the authors upon request.

## Abbreviations

COPD: Chronic Obstructive Pulmonary Disease
DEP: Diesel Exhaust Particles
WNT: Wingless/Int
NAC: N-acetyl cysteine
PM: Particulate Matter
ROS: Reactive Oxygen Species
SPC: Surfactant Protein C
ACT: Acetylated-alpha tubulin
PCA: Principal Component Analysis
GSEA: Gene Set Enrichment Analysis
FDR: False Discovery Rate
qRT-PCR: quantitative Real Time Polymerase Chain Reaction
SEM: Standard error of mean
RAGE: Receptor for Advanced Glycation End Products
CSE: Cigarette Smoke Extract
AhR: Aryl hydrocarbon Receptor
