# Supplementary material for: Diesel exhaust particles distort lung epithelial progenitors and their fibroblast niche
Source: Environ Pollut. Author manuscript; Available in PMC 2024 Jul 16. (PMC11251497; doi:10.1016/j.envpol.2022.119292)
Supplement: Appendix [file NIHMS2004175-supplement-Appendix.docx]

*Appendix A*

**Diesel exhaust particles distort the lung epithelial progenitors and their fibroblast niche**

Xinhui Wu^* a,b^, Chiara Ciminieri^* a,b^, I. Sophie T. Bos ^a,b^, Manon E. Woest ^a,b,c^ , Angela D’Ambrosi ^a,b^, René Wardenaar ^d^, Diana C.J. Spierings ^d^, Melanie Königshoff ^e^, Martina Schmidt ^a,b^, Loes E.M. Kistemaker ^a,b,c^, Reinoud Gosens^#^ ^a,b,c^

^a^ Department of Molecular Pharmacology, Faculty of Science and Engineering, University of Groningen, Antonius Deusinglaan 1, 9713AV, Groningen, The Netherlands

^b^ Groningen Research Institute for Asthma and COPD, University Medical Center Groningen, University of Groningen, Groningen, The Netherlands

^c^ Aquilo BV, Antonius Deusinglaan 1, 9713AV, Groningen, The Netherlands

^d^ European Research Institute for the Biology of Ageing (ERIBA), University of Groningen, University Medical Center Groningen, 9713AV Groningen, The Netherlands

^e^ Division of Pulmonary, Allergy and Critical Care Medicine, Department of Medicine, University of Pittsburgh, Pittsburgh, USA

*Both authors contributed equally and can be cited in either order

#Corresponding author. Department of Molecular Pharmacology, Faculty of Science and Engineering, University of Groningen, Antonius Deusinglaan 1, 9713AV, Groningen, The Netherlands. *E-mail address*: r.gosens@rug.nl

**Extended Materials and Methods**

1. *Reagents preparation*

A stock solution from the diesel particulate matter (NIST 2975, Sigma-Aldrich Chemie N.V.) in culture medium was prepared according to the manufacturer’s instructions. Briefly, the diesel particulate (DEP) was added to the culture medium; to dissolve the particles, this solution was vortexed for 5 min, and then sonicated using Bransonic ultrasonic cleaner (Branson 2510) at 37 °C for 1 h. The DEP stock solution was stored at 4 °C and vortexed before every use. DEP treatments were prepared fresh each time using concentrations of 50, 100, or 200 μg/mL DEP in the organoids and A549 cell cultures.

1. *Cell culture*

Mouse fibroblasts (Mlg [CCL206], ATCC) were cultured in DMEM/F12 medium supplemented with 10% (v/v) fetal bovine serum, 100 U/mL penicillin/ streptomycin, 2 mM L-glutamine, and 1% amphotericin B within a humid atmosphere under 5% CO2 at 37 °C as previously described (Wu et al., 2019b). For organoid experiments, fibroblasts were proliferation-inactivated by incubation in mitomycin C (10 μg/mL, M4287, Sigma) for 2 h, washed 3 times with PBS, and then trypsinized before use in the organoid co-cultures.

A549 adenocarcinoma lung cells (ATCC) were cultured in RPMI medium supplemented with 10% (v/v) fetal bovine serum, 100 U/mL penicillin/ streptomycin, 2 mM L-glutamine, and 1% amphotericin B at 37 °C and 5% CO2. Before treatment, cells were put in starvation medium (0.5% FBS) for 24 hours.

1. *Human organoids*

For human organoids, adult human donor tissue was isolated from histologically normal regions of lung tissue specimens obtained at University Medical Centre Groningen (Groningen, The Netherlands). Human lung tissues were incubated and homogenized overnight in an enzyme mixture at 4 °C; the EpCAM⁺ isolation process was similar to what already described for murine lung tissue; the isolated cells were seeded with human MRC-5 fetal lung fibroblasts (ATCC) in organoid culture. Cultures were incubated in a humid atmosphere under 5% CO2 at 37 °C; fresh medium and treatments were replaced every 2–3 days.

1. *Immunofluorescence staining*

The immunofluorescence staining assay for organoids was performed as described previously by our group (Ng-Blichfeldt et al., 2019; Wu et al., 2019b), with minor modifications. After 14 days in culture, organoids were fixed in acetone diluted 1:1 (v/v) with methanol for 15 min at –20 °C. After fixation, one mL of PBS with 0.02 % sodium azide was added to the well beneath the insert. The organoids were kept at 4 °C for one week after fixation. 5% BSA in PBS was added on top of the inserts to block aspecific sites at room temperature (RT) for 2 hours. Afterwards, primary antibody incubation (anti- pro-SPC, Abcam AB3786; anti- acetylated alpha tubulin, Santa Cruz Technology, sc-23950) was performed in PBS with 0.1% BSA and 0.1% Triton X-100 overnight at 4 °C. The next day, the organoids were washed three times with PBS for 30 min each time, and incubated with secondary antibody for 2h at RT, followed by washing with PBS for 15 min. The organoids on the insert membrane were transferred to a glass slide with two drops of the mounting medium containing DAPI (104139, Abcam), and a coverslip was applied; the slides were kept at 4 °C. Confocal images were acquired using a Leica SP8 microscope at 63x magnification or a Leica DM4000B microscope. Images were obtained with LASX software (open resource, Leica Microsystems GmbH).

1. *Organoid resorting to regain fibroblasts and epithelial cells*

For organoids resorting, 200,000 Epcam^+^ cells and 200,000 CCL-206 fibroblasts were seeded in 1 mL mixture of Matrigel prediluted 1:1 (v/v) with DMEM (supplemented with 10% FBS) in one well of a 6-well plate. After the Matrigel was solidified (> 1 h), 2 mL of organoid culture medium was added on top of the Matrigel. After 7 days culture, 1 mL filtered Dispase (354235, Corning) was added to each well for 30 mins at 37 °C to dissociate the Matrigel. 3 mL MACS buffer (premixed with BSA, 130-091-376, and MACS rinsing solution, 130-091-222, Miltenyi Biotec) was added to stop dispase activity. Organoids were collected and centrifuged at 300g for 5 min. The pellets were resuspended in 5 mL diluted trypsin (1:5, v/v) for 5 min at 37°C, after which 9 mL DMEM/F-12 supplemented with 10% FBS was added to neutralize trypsin action. After centrifugation at 300g for 5 min, cell pellets were incubated with CD326 microbeads for 20 min and resuspended in 8 mL MACS buffer. Cell suspensions were introduced to the QuadroMACS™ Separator system, rendering CD326^-^ fibroblasts and CD326^+^ (Epcam^+^) epithelial cells derived from organoids. These cells were used for further experimentation.

1. *RNA extraction, qRT-PCR and RNA sequencing (RNA seq) analysis*

Total RNA was extracted from A549 cells using TRIzol™ (Life Technologies) for qRT-PCR analysis. RNA concentration and quality were assessed by Nanodrop spectrophotometer (ThermoFisher Scientific) and RNA was converted into cDNA using the Reverse Transcription System (Promega). qRT-PCR was performed with the QuantStudio™ 7 Flex Real-Time PCR Systems (Thermo Fisher Scientific) using FastStart Universal SYBR Green Master (Roche Applied Science). The cycle parameters used in real-time PCR system were denaturation at 95 °C for 30 s, annealing at 59 °C for 30 s and extension at 72 °C for 30 s for 40 cycles, followed by 5 min at 72 °C. qRT-PCR results were analyzed using the 2^-ΔΔCt method (Livak and Schmittgen, 2001).

Total RNA was extracted from cells re-sorted from organoids using NucleoSpin® RNA kit (740955, MACHEREY-NAGEL) according to the manufacturer’s instructions. RNA concentrations and quality were assessed by Nanodrop spectrophotometer and Bioanalyzer system (Agilent). Transcriptome analysis was performed in collaboration with the European Research Institute for the Biology of Ageing (ERIBA, Groningen, The Netherlands). Total RNA (400 ng) was used as input for poly(A) enrichment using NEXTflex Poly(A) Beads followed by library preparation using NEXTflex Rapid Directional qRNA-seq Kit (PerkinElmer). Paired-end sequencing was performed on an Illumina NextSeq 500 system (Illumina; read-1 up to 74 cycles and read-2 up to 9 cycles). The quality of the data was assessed using FastQC (Andrews, 2010). Principal component analyses were performed in R using the R package DESeq2 (Love et al., 2014) in order to visualize the overall effect of experimental covariates as well as batch effects (function: plotPCA). The same R package was used to identify differentially expressed genes between treated and non-treated samples following standard normalization procedures. Genes (transcripts) with an adjusted p-value below 0.05 were considered differentially expressed. Gene set enrichment analysis (GSEA) of the top 500 differentially regulated genes was performed with the Molecular Signatures Database provided by the Broad Institute (<http://www.gsea-msigdb.org/gsea/msigdb/annotate.jsp>) (Subramanian et al., 2005); Wikipathways was used as reference database, and the statistically significant pathway enrichments with FDR q value < 0.05 are reported.

For correlation analysis, we calculated the z-scores of genes upregulated in the *Nrf2 pathway* in our Epcam^+^ control and DEP-treated samples. We then performed a DeSeq2 analysis in R on the Epcam^+^ dataset as previously described, using the z-score as metadata, and extracted the genes included in the *WNT signaling and pluripotency* pathway downregulated in our dataset.

The Gene Expression Omnibus (GEO) data repository (<https://www.ncbi.nlm.nih.gov/geo/>) was used to find transcriptomics datasets from *in vivo* experiments. The dataset GSE22357 was chosen and differential expression analysis was performed with the online tool GEO2R between N-DEP-treated mice and saline-treated control mice samples. GSEA was performed on genes with p-value below 0.05 using MSigDb as previously described.

1. *Murine precision-cut lung slices (PCLS)*

Precision-cut lung slices were prepared as described previously (Wu et al., 2019a). Briefly, C57BL/6J mice lungs were inflated with a low melting-point agarose solution and put on ice to allow the agarose to solidify, before being harvested and separated into lobes. A tissue slicer (Leica VT 1000 S Vibrating blade microtome, Leica Biosystems B.V.) was used to cut lung slices with a thickness of 250 μm. The lung slices were cultured at 37°C and 5% CO2, treated for 24 hours with DEP (200 µg/ml) and then collected for RNA extraction and qRT-PCR analysis as described before.

**References**

Andrews, S., 2010. FastQC: A Quality Control Tool for High Throughput Sequence Data [Online] [WWW Document]. URL https://www.bioinformatics.babraham.ac.uk/projects/fastqc/ (accessed 1.16.22).

Livak, K.J., Schmittgen, T.D., 2001. Analysis of relative gene expression data using real-time quantitative PCR and the 2-ΔΔCT method. Methods 25, 402–408. https://doi.org/10.1006/meth.2001.1262

Love, M.I., Huber, W., Anders, S., 2014. Moderated estimation of fold change and dispersion for RNA-seq data with DESeq2. Genome Biol. 15, 1–21. https://doi.org/10.1186/s13059-014-0550-8

Ng-Blichfeldt, J.P., de Jong, T., Kortekaas, R.K., Wu, X., Lindner, M., Guryev, V., Hiemstra, P.S., Stolk, J., Königshoff, M., Gosens, R., 2019. Tgf-β activation impairs fibroblast ability to support adult lung epithelial progenitor cell organoid formation. Am. J. Physiol. - Lung Cell. Mol. Physiol. 317, L14–L28. https://doi.org/10.1152/ajplung.00400.2018

Subramanian, A., Tamayo, P., Mootha, V.K., Mukherjee, S., Ebert, B.L., Gillette, M.A., Paulovich, A., Pomeroy, S.L., Golub, T.R., Lander, E.S., Mesirov, J.P., 2005. Gene set enrichment analysis: A knowledge-based approach for interpreting genome-wide expression profiles. Proc. Natl. Acad. Sci. 102, 15545–15550. https://doi.org/10.1073/PNAS.0506580102

Wu, X., van Dijk, E.M., Bos, I.S.T., Kistemaker, L.E.M., Gosens, R., 2019a. Mouse Lung Tissue Slice Culture. Methods Mol. Biol. 1940, 297–311. https://doi.org/10.1007/978-1-4939-9086-3_21

Wu, X., van Dijk, E.M., Ng-Blichfeldt, J.P., Bos, I.S.T., Ciminieri, C., Königshoff, M., Kistemaker, L.E.M., Gosens, R., 2019b. Mesenchymal WNT-5A/5B Signaling Represses Lung Alveolar Epithelial Progenitors. Cells 8. https://doi.org/10.3390/cells8101147
