# Supplementary material for: Diesel exhaust particles distort lung epithelial progenitors and their fibroblast niche
Source: Environ Pollut. Author manuscript; Available in PMC 2024 Jul 16. (PMC11251497; doi:10.1016/j.envpol.2022.119292)
Supplement: Supplement [file NIHMS2004175-supplement-Supplement.docx]

*Supplementary tables* *and figures*

**Diesel exhaust particles distort the lung epithelial progenitors and their fibroblast niche**

Xinhui Wu^* a,b^, Chiara Ciminieri^* a,b^, I. Sophie T. Bos^a,b^, Manon E. Woest ^a,b,c^ , Angela D’Ambrosi ^a,b^, René Wardenaar ^d^, Diana C.J. Spierings ^d^, Melanie Königshoff^e^, Martina Schmidt ^a,b^, Loes E.M. Kistemaker ^a,b,c^, Reinoud Gosens^#^ ^a,b,c^

^a^ Department of Molecular Pharmacology, Faculty of Science and Engineering, University of Groningen, Antonius Deusinglaan 1, 9713AV, Groningen, The Netherlands

^b^Groningen Research Institute for Asthma and COPD, University Medical Center Groningen, University of Groningen, Groningen, The Netherlands

^c^ Aquilo BV, Antonius Deusinglaan 1, 9713AV, Groningen, The Netherlands

^d^ European Research Institute for the Biology of Ageing (ERIBA), University of Groningen, University Medical Center Groningen, 9713AV Groningen, The Netherlands

^e^Division of Pulmonary, Allergy and Critical Care Medicine, Department of Medicine, University of Pittsburgh, Pittsburgh, USA

*Both authors contributed equally and can be cited in either order

#Corresponding author. Department of Molecular Pharmacology, Faculty of Science and Engineering, University of Groningen, Antonius Deusinglaan 1, 9713AV, Groningen, The Netherlands. *E-mail address*: r.gosens@rug.nl

|  | **Overlap (# of genes)** | **Genes** |
| --- | --- | --- |
| Upregulated | 269 | Aamp, Abcb8, Abcc3, Abhd4, Acaa1a, Acads, Actb, Actg1, Adgre5, Aes, Ager, Agpat3, Ahnak2, Alas1, Aldh3a1, Aldoa, Amdhd2, Amfr, Ano8, Anxa1, Anxa11, Anxa2, Anxa5, Anxa7, Ap1s1, Apbb1, Aprt, Arf3, Arhgap1, Arhgdia, Arrdc1, Atg7, Atp6ap1, AU021092, Bag6, Bax, BC004004, Bcl2l1, C130074G19Rik, Calm3, Capns1, Cbarp, Cbx6, Ccdc9, Ccnd1, Cd151, Cd36, Cdipt, Cebpa, Cers2, Cfl1, Chmp1a, Chpf2, Cirbp, Cldn18, Clic1, Clic5, Cmtm3, Cndp2, Cnn2, Cnppd1, Cops8, Cpm, Creb3, Cryab, Ctdsp1, Ctnnb1, Ctsa, Ctsc, Ctsd, Ctse, Ctsh, Cuta, Cxcl15, Cyb5r3, Cyp1b1, Cystm1, Cyth2, Dad1, Dazap2, Dcxr, Ddb1, Dnajc5, Dpp3, Drap1, Ehd2, Eif1, Eif3b, elements, Eml2, Emp2, Endod1, Eno1, Epb41l1, Ephx1, Epn1, Eps8l1, F11r, Fads3, Fam20c, Fam234a, Fbrs, Fkbp8, Flna, Gabarap, Galk2, Gipc1, Glmp, Gnai2, Gnas, Gnb2, Gpc1, Gprc5a, Gpx1, Grn, Gsdme, Gsn, Gsta3, Gsto1, Gys1, H13, H2-D1, Hdac5, Hdac7, Ifit1bl1, Il4ra, Kcnk2, Kcnn3, Kctd10, Kif1c, Lgals3, Liph, Llgl2, Lmna, Lmo7, Lpcat4, Lxn, Ly6g6c, Lypla2, Lzts2, Mal, Map4, Map7d1, Mbd6, Mfge8, Mfn2, Mfsd1, Mgat4b, Mgrn1, Mlf2, Msln, Mxd1, Myadm, Myh14, Myh7, Myl6, Naaa, Nbeal2, Necap2, Nedd8, Nfkb2, Npdc1, Nqo1, Nt5e, Ocln, Ogdh, Osgin1, P4hb, Pam, Pawr, Pfkl, Pfn1, Pgd, Pkm, Plaur, Plin2, Plscr3, Plxnb2, Plxnd1, Polr2e, Ppp1r16a, Ppp2r1a, Prr15l, Prss8, Psapl1, Psmd8, Ptms, Ptp4a3, R3hdm4, Rab11b, Rab1b, Rab5c, Rac1, Rap1gap, Rbm3, Rdh10, Rela, Renbp, Rhoa, Ripk3, Rnf5, Rnh1, S100a14, S100a16, S100a6, Scgb1a1, Sectm1b, Selenof, Selenos, Sema3c, Serpinb6a, Sftpa1, Sftpd, Slc16a3, Slc24a3, Slc25a10, Slc25a24, Slc25a39, Slc25a4, Slc35e4, Slc39a8, Slc48a1, Slc4a2, Slc6a14, Slc9a2, Slc9a4, Spryd3, Sqstm1, Src, Ssbp3, St3gal5, Stk11ip, Stub1, Sumf1, Sun2, Syngr2, Szrd1, Tagln2, Taldo1, Tapbp, Tcf25, Tesk1, Tex264, Tgm1, Timp2, Tinagl1, Tmbim6, Tmem176a, Tmem176b, Tmem214, Tmem63b, Tmprss2, Tmsb4x, Tnip3, Tpi1, Triobp, Tspan3, Uba1, Ube2m, Ubl5, Unc13d, Usp5, Vat1, Vcam1, Vcp, Wbp1l, Wdr1, Wfs1, Xdh, Yipf3 |
| Downregulated | 162 | 2010111I01Rik, 2610203C20Rik, 4932438A13Rik, 4933431E20Rik, Adamts1, Add2, Ago3, Ahnak, Akap9, Amn1, Ankrd1, Ankrd10, Apc, Arid5b, Atf7ip, Atrx, Banp, Birc6, Brwd1, Caprin2, Cbl, Ccnt1, Cd80, Cdk6, Cmah, Col8a1, Cpne8, Cspp1, D10Wsu102e, Dgkh, Diaph2, Dst, Dut, E2f8, Eci2, Egfr, Eif4a2, elements, Eml4, Ermp1, Etv1, Fam110c, Fam126b, Fam135a, Fastkd1, Fmn1, Fmo2, Gja1, Gm38394, Gm42664, Hif1a, Hivep2, Hlf, Hoxb4, Hspa1a, Hspa1b, Huwe1, Ifi27, Igfbp5, Il6st, Itgb3, Itpr2, Jun, Kcnq1ot1, Kif5b, Kmt2a, Ktn1, Lman1, Lox, Lpcat2, Lpp, Lrp1, Macf1, Man1a, Mapk6, Mast4, Mbtd1, Mfap3l, Mib1, Muc16, Mycbp2, Naa25, Nav1, Nav2, Ncoa6, Nedd9, Nemf, Nf1, Nfat5, Nfib, Nktr, Nrip1, Nup88, P2rx2, Pan3, Pde7a, Pdgfrl, Phip, Phka2, Pla2g7, Plod2, Pmepa1, Prpf39, Prrc2c, Ptbp2, Pxdn, R3hdm1, Rbms3, Rbp1, Reg3g, Rexo5, Rgs4, Rnd3, Rock2, Rprd1a, Setd5, Sfi1, Skap2, Skil, Slc16a7, Slc25a36, Slc25a37, Slc39a10, Slc40a1, Slc4a7, Slc5a3, Slitrk6, Snhg8, Snrnp48, Snx27, Sos1, Sox9, Sp1, Srek1, Steap2, Stxbp5, Sulf1, Taf1d, Tcf4, Tcim, Tead1, Tgif1, Thoc2, Tmeff1, Tnc, Tnnt2, Trp53bp1, Ube3a, Ubn2, Uprt, Vamp1, Vps37a, Wnk1, Wsb1, Zcchc11, Zfp318, Zfp329, Zfp462, Zfp52, Zfp644, Zfp809, Zfp871, Zufsp |

**Suppl. Table 1.** List of genes that are commonly up- and down-regulated in both epithelial and fibroblasts population after DEP exposure (DeSeq2 analysis). *Related to Fig.2.*

**Suppl. Table 2.** Pathway enrichment analysis from upregulated genes in the fibroblasts population (Gene Set Enrichment Analysis, MSigDB, FDR q value <0.05, Wikipathways gene set). *Related to Fig. 3.*

| **# Genes in Gene Set (K)** | **Gene Set Name** | **# Genes in Overlap (k)** | **k/K** | **p-value** | **FDR q-value** | **Genes** |
| --- | --- | --- | --- | --- | --- | --- |
| 438 | Wp_vegfavegfr2_signaling_pathway | 30 | 0.0685 | 3.83E-14 | 2.35E-11 | Ctnnb1; Rac1; Rhoa; Ocln; Rplp2; Eif3h; Psmd4; Tnxb; Tkt; Ldha; Gpx1; Rhoc; Prdx2; Ap2a1; Ap2s1; Akt1s1; Calr; Plaur; Taok2; Pfn1; Hdac5; Eif4g2; Gipc1; Clic1; Snd1; Lrrc59; Ptma; Ssr4; Slc25a11; Fxr2 |
| 219 | Wp_ciliary_landscape | 16 | 0.0731 | 1.46E-08 | 4.35E-06 | Ctnnb1; Rac1; Mcm3; Mcm2; Mcm5; Ube2d2; Vim; Ddx5; H3-3a; Htra2; Dynll2; Cops8; Iqgap3; Myl6; Ctsa; Ssna1; Rhoa |
| 55 | Wp_pathogenic_escherichia_coli_infection | 9 | 0.1636 | 2.12E-08 | 4.35E-06 | Ctnnb1; Rhoa; Ocln; Actb; Tuba1b; Tubb4b; Tubb; Arpc1b; Arpc3 |
| 90 | Wp_cytoplasmic_ribosomal_proteins | 10 | 0.1111 | 1.57E-07 | 2.42E-05 | Rplp2; Rpl35; Rpl7a; Rpl18; Rpl37a; Rps4x; Rps9; Rps14; Rps15a; Rps21 |
| 53 | Wp_translation_factors | 8 | 0.1509 | 2.51E-07 | 3.09E-05 | Eif3h; Eif4a1; Eef1a1; Eif1; Eef1b2; Eef1d; Eif5a; Eif6 |
| 279 | Wp_il18_signaling_pathway | 16 | 0.0573 | 4.09E-07 | 4.20E-05 | Ctnnb1; Bax; Spp1; Col1a2; Eno1; Tnfrsf1a; Acta2; Rxrb; Cd36; Tnip3; Cd81; Lars2; Grn; Ccdc9; Myh7; Nrn1 |
| 42 | Wp_dna_replication | 7 | 0.1667 | 7.11E-07 | 6.25E-05 | Mcm3; Mcm2; Mcm5; Pole; Prim1; Rfc5; Pold1 |
| 64 | Wp_g1_to_s_cell_cycle_control | 8 | 0.125 | 1.11E-06 | 7.61E-05 | Mcm3; Mcm2; Mcm5; Pole; Prim1; Cdk1; Ccnd3; Atf6b |
| 64 | Wp_proteasome_degradation | 8 | 0.125 | 1.11E-06 | 7.61E-05 | Ube2d2; Psmd4; Ube2d3; Hla-b; Hla-a; Psmb6; Rpn1; Rpn2 |
| 145 | Wp_nrf2_pathway | 11 | 0.0759 | 1.83E-06 | 1.13E-04 | Rxra; Ppard; Slc39a8; Nqo1; Abcc3; Aldh3a1; Ager; Slc39a13; Gsta3; Slc6a14; Slc6a8 |
| 321 | Wp_nuclear_receptors_metapathway | 16 | 0.0498 | 2.57E-06 | 1.44E-04 | Rxra; Ppard; Slc39a8; Nqo1; Abcc3; Aldh3a1; Ager; Slc39a13; Gsta3; Slc6a14; Slc6a8; Cdk1; Bax; Acaa1; Ptpa; Cyp1b1 |
| 202 | Wp_focal_adhesion | 12 | 0.0594 | 7.99E-06 | 4.10E-04 | Ccnd3; Ctnnb1; Spp1; Col1a2; Rhoa; Actb; Rac1; Tnxb; Flna; Myl9; Pdgfa; Vasp |
| 88 | Wp_retinoblastoma_gene_in_cancer | 8 | 0.0909 | 1.25E-05 | 5.93E-04 | Ccnd3; Cdk1; Mcm3; Pole; Prim1; Rfc5; Top2a; Mapk13 |
| 44 | Wp_metabolic_reprogramming_in_colon_cancer | 6 | 0.1364 | 1.51E-05 | 6.63E-04 | Eno1; Tkt; Ldha; Taldo1; Mdh2; Slc16a3 |
| 160 | Wp_nonalcoholic_fatty_liver_disease | 10 | 0.0625 | 2.93E-05 | 1.20E-03 | Rac1; Rxra; Bax; Tnfrsf1a; Ndufc2; Ndufv1; Cox8a; Cox6b1; Cebpa; Cyc1 |
| 133 | Wp_mrna_processing | 9 | 0.0677 | 3.93E-05 | 1.51E-03 | Hnrnpd; Prmt1; Ybx1; Fus; Sf3b2; Hnrnpm; Snrpb; Pabpn1; Sf3a2 |
| 106 | Wp_electron_transport_chain_oxphos_system_in_mitochondria | 8 | 0.0755 | 4.86E-05 | 1.76E-03 | Ndufc2; Ndufv1; Cox8a; Cox6b1; Ucp2; Atp5f1b; Atp5mc3; Slc25a5 |
| 7 | Wp_pentose_phosphate_metabolism | 3 | 0.4286 | 6.06E-05 | 2.07E-03 | Tkt; Taldo1; Pgls |
| 86 | Wp_pyrimidine_metabolism | 7 | 0.0814 | 8.94E-05 | 2.77E-03 | Pole; Prim1; Pold1; Tk1; Polr1c; Polr2j; Nt5c |

**Suppl. Table 3**. Pathway enrichment analysis from downregulated genes in the fibroblasts population (Gene Set Enrichment Analysis, MSigDB; FDR q value <0.05, Wikipathways gene set). *Related to Fig. 3.*

| **# Genes in Gene Set (K)** | **Gene Set Name** | **# Genes in Overlap (k)** | **k/K** | **p-value** | **FDR q-value** | **Genes** |
| --- | --- | --- | --- | --- | --- | --- |
| 131 | Wp_adipogenesis | 11 | 0.084 | 4.26E-07 | 2.62E-04 | Ppargc1a; Ncor1; Rora; Sp1; Mef2a; Twist1; Igf1; Hif1a; Il6st; Gadd45a; Trib3 |
| 37 | Wp_factors_and_pathways_affecting_insulinlike_growth_factor_igf1akt_signaling | 6 | 0.1622 | 4.11E-06 | 1.26E-03 | Ppargc1a; Igf1; Igfbp5; Wasl; Jkamp; Tnfsf9 |
| 158 | Wp_myometrial_relaxation_and_contraction_pathways | 10 | 0.0633 | 1.78E-05 | 1.95E-03 | Igfbp5; Sp1; Jun; Gng12; Rgs2; Gnb5; Camk2d; Itpr2; Rgs17; Rgs4 |
| 438 | Wp_vegfavegfr2_signaling_pathway | 17 | 0.0388 | 1.85E-05 | 1.95E-03 | Jun; Rock2; Itgb3; Hspa1a; Igfbp7; Fhl2; Herpud1; Mmp2; Nr4a2; Mmp10; Afdn; Dpm1; Prrc2c; Brd4; Gigyf2; Tbca; Eea1 |
| 309 | Wp_focal_adhesionpi3kaktmtorsignaling_pathway | 14 | 0.0453 | 1.88E-05 | 1.95E-03 | Itgb3; Gng12; Ppargc1a; Igf1; Hif1a; Sos1; Tnc; Itgb8; Lama2; Fgf11; Gng10; Ppp2r5a; Strada; Col11a1 |
| 72 | Wp_mecp2_and_associated_rett_syndrome | 7 | 0.0972 | 2.12E-05 | 1.95E-03 | Igf1; Sp1; Ncor1; Ube3a; Gad1; Cdon; Tet2 |
| 163 | Wp_egfegfr_signaling_pathway | 10 | 0.0613 | 2.33E-05 | 1.95E-03 | Sp1; Sos1; Jun; Mef2a; Twist1; Sh3gl3; Asap1; Plscr1; Ptpn12; Pxdn |
| 133 | Wp_tgfbeta_signaling_pathway | 9 | 0.0677 | 2.76E-05 | 1.95E-03 | Sp1; Sos1; Jun; Mef2a; Itgb3; Tnc; Runx2; Zeb2; Zeb1 |
| 321 | Wp_nuclear_receptors_metapathway | 14 | 0.0436 | 2.86E-05 | 1.95E-03 | Sp1; Jun; Ppargc1a; Hspa1a; Rgs2; Gstm3; Il11; Nrg1; Fth1; Ggt1; Slc39a10; Slc5a3; Smarca1; Ankrd1 |
| 108 | Wp_senescence_and_autophagy_in_cancer | 8 | 0.0741 | 4.03E-05 | 2.48E-03 | Jun; Igf1; Igfbp7; Igfbp5; Il6st; Kmt2a; Inhba; Rb1cc1 |
| 18 | Wp_mfap5_effect_on_permeability_and_motility_of_endothelial_cells_via_cytoskeleton_rearrangement | 4 | 0.2222 | 4.86E-05 | 2.72E-03 | Jun; Itgb3; Mfap5; Lpp |
| 91 | Wp_androgen_receptor_signaling_pathway | 7 | 0.0769 | 9.63E-05 | 4.93E-03 | Jun; Sp1; Runx2; Ncor1; Ube3a; Rock2; Fhl2 |
| 207 | Wp_circadian_rhythm_related_genes | 10 | 0.0483 | 1.72E-04 | 8.15E-03 | Jun; Ncor1; Ube3a; Rock2; Kmt2a; Ppargc1a; Rora; Clock; Thrap3; Ogt |
| 49 | Wp_exerciseinduced_circadian_regulation | 5 | 0.102 | 2.61E-04 | 1.15E-02 | Clock; Gstm3; Herpud1; Tab2; Pura |
| 13 | Wp_mfap5mediated_ovarian_cancer_cell_motility_and_invasiveness | 3 | 0.2308 | 4.10E-04 | 1.68E-02 | Jun; Itgb3; Mfap5 |
| 31 | Wp_cell_migration_and_invasion_through_p75ntr | 4 | 0.129 | 4.44E-04 | 1.71E-02 | Jun; Twist1; Mmp2; Kidins220 |
| 202 | Wp_focal_adhesion | 9 | 0.0446 | 6.47E-04 | 2.34E-02 | Jun; Itgb3; Rock2; Igf1; Sos1; Tnc; Itgb8; Lama2; Tln1 |
| 164 | Wp_epithelial_to_mesenchymal_transition_in_colorectal_cancer | 8 | 0.0488 | 7.11E-04 | 2.43E-02 | Sos1; Twist1; Mmp2; Zeb2; Zeb1; Hif1a; Eed; Nubpl |

**Suppl. Table 4**. Pathway enrichment analysis from upregulated genes in the epithelial (Epcam^+^) population (Gene Set Enrichment Analysis, MSigDB; FDR q value <0.05, Wikipathways gene set). *Related to Fig. 3.*

| **# Genes in Gene Set (K)** | **Gene Set Name** | **# Genes in Overlap (k)** | **k/K** | **p-value** | **FDR q-value** | **Genes** |
| --- | --- | --- | --- | --- | --- | --- |
| 321 | Wp_nuclear_receptors_metapathway | 41 | 0.1277 | 4.2E-30 | 2.58E-27 | Cyp1b1; Nqo1; Nfe2l2; Tnf; Ahrr; Aldh3a1; Gpx3; Gstt2; Gsta5; Gsta3; Mgst3; Cyp3a4; Cyp2b6; Gclc; Ptgs2; Sod3; Abcc3; Ces1; Srxn1; Cbr1; Slc7a11; Slc2a3; Slc39a8; Pgd; Ptgr1; Abcc5; Ager; Slc5a5; Slc6a20; Slc2a6; Slc6a14; Cbr3; Ccl20; Angptl4; Slc26a2; Serpinb9; Scnn1a; Cavin2; Ehhadh; Alas1; Pdk4 |
| 145 | Wp_nrf2_pathway | 26 | 0.1793 | 2.18E-23 | 6.71E-21 | Nqo1; Nfe2l2; Aldh3a1; Gpx3; Gstt2; Gsta5; Gsta3; Mgst3; Gclc; Sod3; Abcc3; Ces1; Srxn1; Cbr1; Slc7a11; Slc2a3; Slc39a8; Pgd; Ptgr1; Abcc5; Ager; Slc5a5; Slc6a20; Slc2a6; Slc6a14; Cbr3 |
| 48 | Wp_aryl_hydrocarbon_receptor_netpath | 8 | 0.1667 | 7.56E-08 | 1.55E-05 | Nqo1; Nfe2l2; Gclc; Cyp1b1; Tnf; Ahrr; Ptgs2; Ret |
| 34 | Wp_oxidative_stress | 7 | 0.2059 | 1.08E-07 | 1.67E-05 | Nqo1; Nfe2l2; Gclc; Gpx3; Gstt2; Sod3; Maoa |
| 23 | Wp_photodynamic_therapyinduced_nfe2l2_nrf2_survival_signaling | 6 | 0.2609 | 1.97E-07 | 2.43E-05 | Nqo1; Nfe2l2; Gclc; Abcc3; Ces1; Srxn1 |
| 185 | Wp_metapathway_biotransformation_phase_i_and_ii | 13 | 0.0703 | 2.86E-07 | 2.93E-05 | Gpx3; Gstt2; Cyp1b1; Gsta5; Gsta3; Mgst3; Cyp3a4; Cyp2b6; Cyp2s1; Cyp4f22; Chst4; Akr1b10; Hs3st3b1 |
| 68 | Wp_sarscov2_innate_immunity_evasion_and_cellspecific_immune_response | 8 | 0.1176 | 1.21E-06 | 1.06E-04 | Tnf; Cxcl3; Cxcl1; Stat1; Ace2; Mx1; Ifit2; Cxcl6 |
| 21 | Wp_simplified_interaction_map_between_loxl4_and_oxidative_stress_pathway | 5 | 0.2381 | 3.53E-06 | 2.71E-04 | Nqo1; Nfe2l2; Fgf7; Anxa5; Bmp2 |
| 150 | Wp_regulation_of_actin_cytoskeleton | 10 | 0.0667 | 1.07E-05 | 7.33E-04 | Fgf7; Tmsb4x; Gsn; Fgf18; Rac1; Rac2; Rras; Mylk; Pip5k1b; Cyfip2 |
| 45 | Wp_prostaglandin_synthesis_and_regulation | 6 | 0.1333 | 1.28E-05 | 7.90E-04 | Anxa5; Ptgs2; Cbr1; Pparg; Anxa1; Scgb1a1 |
| 71 | Wp_glucocorticoid_receptor_pathway | 7 | 0.0986 | 1.85E-05 | 9.63E-04 | Ptgs2; Ccl20; Angptl4; Slc26a2; Serpinb9; Scnn1a; Cavin2 |
| 48 | Wp_aryl_hydrocarbon_receptor_pathway | 6 | 0.125 | 1.88E-05 | 9.63E-04 | Nqo1; Nfe2l2; Tnf; Cyp1b1; Ahrr; Aldh3a1 |
| 15 | Wp_phytochemical_activity_on_nrf2_transcriptional_activation | 4 | 0.2667 | 2.18E-05 | 1.03E-03 | Nqo1; Nfe2l2; Gclc; Slc7a11 |
| 279 | Wp_il18_signaling_pathway | 13 | 0.0466 | 2.60E-05 | 1.10E-03 | Tnf; Ptgs2; Ccl20; Tmsb4x; Bmp2; Cxcl3; Ier3; Tnfrsf11b; Il18; Tnip3; Myh7; Abhd16a; Il18rap |
| 32 | Wp_constitutive_androstane_receptor_pathway | 5 | 0.1562 | 3.14E-05 | 1.21E-03 | Cyp3a4; Cyp2b6; Abcc3; Ehhadh; Alas1 |
| 54 | Wp_apoptosisrelated_network_due_to_altered_notch3_in_ovarian_cancer | 6 | 0.1111 | 3.73E-05 | 1.32E-03 | Tnf; Ier3; Nqo1; Gclc; Anxa5; Thbs1 |
| 108 | Wp_senescence_and_autophagy_in_cancer | 8 | 0.0741 | 3.85E-05 | 1.32E-03 | Thbs1; Bmp2; Cxcl1; Gsn; Irf5; Plau; Atg7; Il24 |
| 58 | Wp_tgfbeta_receptor_signaling | 6 | 0.1034 | 5.62E-05 | 1.82E-03 | Thbs1; Tnf; Bmp4; Lif; Stat1; Itgb6 |
| 185 | Wp_vitamin_d_receptor_pathway | 10 | 0.0541 | 6.48E-05 | 1.99E-03 | Irf5; Cyp3a4; Cyp2b6; Tnfrsf11b; Cyp2s1; Timp2; S100a4; Mxd1; Sema3b; S100g |

**Suppl. Table 5.** Pathway enrichment analysis from downregulated genes in the epithelial (Epcam^+^) population (Gene Set Enrichment Analysis, MSigDB; FDR q value <0.05, Wikipathways gene set). *Related to Fig. 3.*

| **# Genes in Gene Set (K)** | **Gene Set Name** | **# Genes in Overlap (k)** | **k/K** | **p-value** | **FDR q-value** | **Genes** |
| --- | --- | --- | --- | --- | --- | --- |
| 81 | Wp_dna_irdamage_and_cellular_response_via_atr | 20 | 0.2469 | 1.50E-13 | 3.21E-11 | Cdk1; Sp1; Foxm1; Chek1; Cdc45; Bard1; Atm; Plk1; Cdc25c; Brca1; Brca2; H2ax; Tp53bp1; Bcl6; Exo1; Fanci; Fanca; Topbp1; Ube2d3; Clspn ; |
| 123 | Wp_hippomerlin_signaling_dysregulation | 24 | 0.1951 | 1.56E-13 | 3.21E-11 | Foxm1; Itgb3; Met; Itgb4; Kdr; Itgb5; Egfr; Igf1r; Itga3; Itga6; Itga10; Bub1b-pak6; Tead4; Tead1; Nf2; Cdh6; Ngfr; Ntrk2; Tead3; Mst1; Prkar2b; Cdh3; Cdh10; Dcaf1 |
| 438 | Wp_vegfavegfr2_signaling_pathway | 45 | 0.1027 | 3.85E-13 | 5.92E-11 | Itgb3; Kdr; Itgb5; Tead4; Jun; Pik3r1; Cav1; Ets1; Prkd1; Bcl2; Actg1; Rock2; Smarca2; Rps6; Rpl13a; Acaca; Plcg1; Nr4a1; Hspb1; Ptprz1; Arnt; Hspa1a; Prdx6; Prkaa2; Nrp2; Adamts1; Eif4e; Ccn1; Gja1; Cbl; Rack1; Slc8a1; Igfbp3; Birc5; Ephb2; Srpk1; Nap1l1; Lman1; Dnajb4; Kank1; Prrc2c; Fjx1; Shroom2; Bmx; Fscn1 |
| 90 | Wp_cytoplasmic_ribosomal_proteins | 20 | 0.2222 | 1.27E-12 | 1.56E-10 | Rps6; Rpl13a; Rps27a; Rpl9; Rpl12; Rpl15; Rpl22; Rpl32; Rpl34; Rpl35a; Rps3; Rps9; Rps10; Rps12; Rps13; Rps14; Rps15a; Rps18; Rps24; Rps25 |
| 133 | Wp_tgfbeta_signaling_pathway | 23 | 0.1729 | 6.97E-12 | 6.12E-10 | Cdk1; Ccnb2; Itgb3; Jun; Pik3r1; Cav1; Ets1; Met; Itgb4; Sp1; Ep300; Sos1; Tnc; Atf3; Rbl1; Fos; Fosb; Skil; Tgif1; Tert; Klf10; Nedd9; Nup153 |
| 122 | Wp_cell_cycle | 22 | 0.1803 | 8.37E-12 | 6.43E-10 | Cdk1; Ccnb2; Ep300; Rbl1; Chek1; Cdc45; Cdk6; Ccna2; Cdc25b; Mcm6; Ttk; Atm; Plk1; Cdc25c; Ccnd2; Orc4; Dbf4; Bub1; Espl1; Cdc20; Cdc14b; Pkmyt1 |
| 279 | Wp_il18_signaling_pathway | 31 | 0.1111 | 2.50E-10 | 1.35E-08 | Ccnb2; Ccna2; Jun; Pik3r1; Sp1; Atf3; Fos; Bcl2; Plcg1; Nr4a1; Hspb1; Ptprz1; Znf219; Timp3; Mmp13; Socs3; Nfatc4; Irf6; Pld1; Rasa3; Tf; Cflar; Arg1; Timp1; Baz1b; Pla2g7; Spon1; Nfkbiz; Bpgm; Tgm2; Slc4a7 |
| 321 | Wp_nuclear_receptors_metapathway | 33 | 0.1028 | 5.10E-10 | 2.24E-08 | Jun; Egfr; Sp1; Cdk1; Ep300; Arnt; Hspa1a; Prdx6; Esr1; Ahr; Cap2; Snai2; Nrip1; Ankrd1; Stom; Edn2; Arl5b; Ktn1; Prrg4; Tns4; Nav3; Fgfbp1; Serpinb2; Slc7a5; Scd; Ces1; Ces2; Gsta1; Slc39a10; Slc5a9; Dnajb1; Slc5a3; Ncoa6 |
| 164 | Wp_epithelial_to_mesenchymal_transition_in_colorectal_cancer | 21 | 0.128 | 1.58E-08 | 5.13E-07 | Snai2; Pik3r1; Sos1; Col4a4; Nrp2; Foxm1; Fzd1; Lrp5; Lrp6; Wnt4; Fzd3; Fzd4; Notch3; Jag2; Hif1a; Dsp; Cldn2; Tmprss4; Fmnl2; Pkp1; Cldn10 |
| 65 | Wp_endochondral_ossification | 13 | 0.2 | 4.22E-08 | 1.20E-06 | Igf1r; Ghr; Timp3; Mmp13; Adamts1; Bmp7; Sox9; Pthlh; Gli3; Enpp1; Sox5; Mgp; Slc38a2 |
| 309 | Wp_focal_adhesionpi3kaktmtorsignaling_pathway | 29 | 0.0939 | 4.28E-08 | 1.20E-06 | Pik3r1; Sos1; Col4a4; Hif1a; Egfr; Itgb3; Met; Itgb4; Tnc; Kdr; Itgb5; Igf1r; Itga3; Itga6; Itga10; Thbs2; Lamb1; Lama1; Rps6; Acaca; Prkaa2; Eif4e; Ngfr; Ghr; Jak3; Osmr; Lipe; Epas1; Tbc1d1 |
| 66 | Wp_oncostatin_m_signaling_pathway | 13 | 0.197 | 5.11E-08 | 1.37E-06 | Timp3; Mmp13; Pik3r1; Sos1; Hif1a; Rps6; Jak3; Osmr; Fos; Socs3; Ccn1; Il6st; Lifr |
| 93 | Wp_corticotropinreleasing_hormone_signaling_pathway | 15 | 0.1613 | 8.13E-08 | 2.05E-06 | Fos; Acaca; Prkaa2; Sp1; Bcl2; Plcg1; Nr4a1; Fosb; Gja1; Tfap2a; Tcf4; Gnai1; Casp12; Cyp21a2; Krt14 |
| 42 | Wp_dna_replication | 10 | 0.2381 | 2.73E-07 | 5.59E-06 | Cdc45; Mcm6; Orc4; Dbf4; Pole; Prim1; Pola1; Pola2; Pold1; Mcm10 |
| 42 | Wp_il2_signaling_pathway | 10 | 0.2381 | 2.73E-07 | 5.59E-06 | Fos; Bcl2; Pik3r1; Sos1; Rps6; Jak3; Socs3; Jun; Ccnd2; Cbl |
| 161 | Wp_insulin_signaling | 18 | 0.1118 | 1.26E-06 | 2.17E-05 | Fos; Pik3r1; Sos1; Socs3; Jun; Cbl; Prkaa2; Igf1r; Enpp1; Eif4e; Lipe; Map3k1; Ptprf; Map3k4; Mapk6; Kif5b; Tbc1d4; Flot1 |
| 86 | Wp_pyrimidine_metabolism | 13 | 0.1512 | 1.27E-06 | 2.17E-05 | Enpp1; Pole; Prim1; Pola1; Pola2; Pold1; Enpp3; Dpyd; Tk1; Cad; Uprt; Ctps1; Dut |
| 163 | Wp_egfegfr_signaling_pathway | 18 | 0.1104 | 1.51E-06 | 2.45E-05 | Fos; Pik3r1; Sos1; Jun; Cbl; Map3k1; Map3k4; Sp1; Plcg1; Fosb; Gja1; Egfr; Cav1; Vav3; Pld1; Aurka; Plce1; Pxdn |
| 185 | Wp_ras_signaling | 19 | 0.1027 | 2.34E-06 | 3.35E-05 | Pik3r1; Sos1; Plcg1; Egfr; Pld1; Plce1; Igf1r; Met; Kdr; Ngfr; Bub1b-pak6; Rasa3; Ets1; Ntrk2; Nf1; Tiam1; Abl2; Rasa4; Rasal2 |
| 185 | Wp_vitamin_d_receptor_pathway | 19 | 0.1027 | 2.34E-06 | 3.35E-05 | Timp3; Hif1a; Pthlh; Lrp5; Cldn2; Nrip1; Slc8a1; Igfbp3; Bcl6; Klf4; Igfbp5; Ephb4; Id4; Cd40; Steap4; Cst6; Serpinb1; Satb1; Cracr2a |
| 91 | Wp_androgen_receptor_signaling_pathway | 13 | 0.1429 | 2.44E-06 | 3.41E-05 | Pik3r1; Egfr; Bub1b-pak6; Jun; Sp1; Cav1; Ep300; Rock2; Tgif1; Rack1; Brca1; Ube3a; Znf318 |
| 106 | Wp_wnt_signaling_pathway_and_pluripotency | 14 | 0.1321 | 2.64E-06 | 3.60E-05 | Jun; Ep300; Lrp5; Ccnd2; Fzd1; Lrp6; Wnt4; Fzd3; Fzd4; Prkd1; Apc; Tcf7; Sox2; Racgap1 |
| 16 | Wp_regulation_of_sister_chromatid_separation_at_the_metaphaseanaphase_transition | 6 | 0.375 | 3.81E-06 | 4.88E-05 | Bub1; Espl1; Cdc20; Cenpe; Mad2l1; Bub1b |


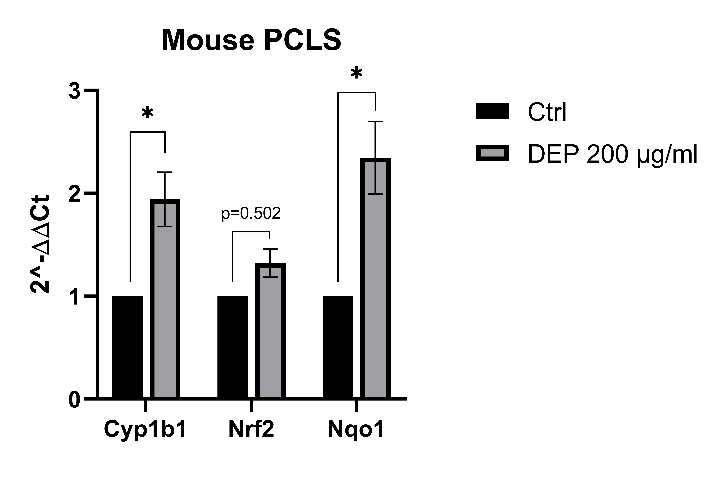
**Suppl. Fig. 1.** Gene expression of cytochrome gene *Cyp1b1*, anti-oxidant genes *Nrf2* and *Nqo1* in murine *ex-vivo* precision-cut lung slices (PCLS) treated with 200 µg/ml DEP for 24 hours, N=3. *p<0.05.
